# Beijing’s central role in global artificial intelligence research

**DOI:** 10.1038/s41598-022-25714-0

**Published:** 2022-12-12

**Authors:** Bedoor AlShebli, Enshu Cheng, Marcin Waniek, Ramesh Jagannathan, Pablo Hernández-Lagos, Talal Rahwan

**Affiliations:** 1 Social Science Division, New York University Abu Dhabi, Abu Dhabi, UAE; 2 Computer Science, Science Division, New York University Abu Dhabi, Abu Dhabi, UAE; 3 Engineering Division, New York University Abu Dhabi, Abu Dhabi, UAE; 4 Sy Syms School of Business, Yeshiva University, New York, USA

**Keywords:** Computational science, Computer science, Scientific data

## Abstract

Nations worldwide are mobilizing to harness the power of Artificial Intelligence (AI) given its massive potential to shape global competitiveness over the coming decades. Using a dataset of 2.2 million AI papers, we study inter-city citations, collaborations, and talent migrations to uncover dependencies between Eastern and Western cities worldwide. Beijing emerges as a clear outlier, as it has been the most impactful city since 2007, the most productive since 2002, and the one housing the largest number of AI scientists since 1995. Our analysis also reveals that Western cities cite each other far more frequently than expected by chance, East–East collaborations are far more common than East–West or West–West collaborations, and migration of AI scientists mostly takes place from one Eastern city to another. We then propose a measure that quantifies each city’s role in bridging East and West. Beijing’s role surpasses that of all other cities combined, making it the central gateway through which knowledge and talent flow from one side to the other. We also track the center of mass of AI research by weighing each city’s geographic location by its impact, productivity, and AI workforce. The center of mass has moved thousands of kilometers eastward over the past three decades, with Beijing’s pull increasing each year. These findings highlight the eastward shift in the tides of global AI research, and the growing role of the Chinese capital as a hub connecting researchers across the globe.

## Introduction

The science of endowing machines with artificial intelligence (AI) has propelled the pace of innovation^1–4^ and introduced positive shifts in various aspects of life, such as labor markets^5–10^, healthcare^11–14^, gender equality^15^, crime reduction^16^, and transportation safety^17–19^, among many others. But AI applications can also lead to adverse outcomes such as discrimination^20–24^, polarization and violence^25–27^, and the use of autonomous weapons^28–30^.

The application of AI continues to grow in scope and sophistication, and as AI raises great opportunities, it also entails significant risks^31,32^. Pundits often speak about countries competing to exert control over the field of AI, but framing AI and its application as technologies that countries must compete for is too narrow a view when the goal is to understand the evolution of AI knowledge. More specifically, AI knowledge, as any other knowledge, builds upon existing knowledge. For example, new AI applications developed worldwide usually build on open source software frameworks created in the U.S., such as TensorFlow (Google), PyTorch (Facebook), or Microsoft CNTK. Thus, our approach is not one of competition but one of dependencies. In this paper, we document dependencies that take the form of networks of AI citations, research collaboration and scientists’ migration.

Our focus is on networks of cities rather than countries, as aggregating knowledge production at the country-level blunts important variation across cities. Cities have always been the engines of innovation and wealth creation as well as the hot spots of production and consumption^33–39^. In our study of cities, we focus on AI research papers due to the role that science plays in stimulating innovation and diffusing knowledge across borders without frictions^40^. Knowledge from academic research is a public good—its consumption does not exclude others, nor reduces the amount available to them. Thus, unlike private goods such as AI-based products or services, knowledge stemming from AI papers should, at least in principle, flow unhindered by tariffs, transportation costs, or other political and economic constraints. Whether AI knowledge flows unhindered in practice is one of the questions we investigate in this paper. More broadly, we set out to address the following questions: How do cities compare in terms of their research output and AI workforce? Does AI research from a given city influence certain regions more than others? How do Eastern cities compare to Western ones? Are there cities that bridge East and West, effectively acting as a bridge through which AI citations, workforce, and collaborations flow between the two sides?

Networks of cities have already been studied in various contexts, including road transportation systems^41^, production of cultural goods^42^, communication patterns^43^, and epidemiology^44,45^. Research in this area revealed how geopolitical considerations shape the communities of cities^46^ and how network of cities create regional synergistic effects^47^. Papers that have studied the scientific activities of cities seek to understand how such activities are related to the city’s population size^48^ or economic complexity^49^. In contrast, we focus on the scientific activities of cities in the context of AI with a particular emphasis on East vs. West. AI bibliometrics have already attracted attention in the Science of Science literature. For example, Frank et al.^50^ examined the academic disciplines that frequently cite or get cited by AI papers, Tang et al.^51^ and Ahmed and Wahed^52^ analyzed patterns of citations between academic and industrial research, Tang et al.^53^ analyzed the rate of growth in AI papers and AI scientists using AI papers from arXiv.org (a popular repository of preprints), Bianchini et al.^54^ studied the effect of new techniques (such as Deep Learning) on practical outcomes (health care), while Martínez-Plumed et al.^55^ studied the consolidation of AI research communities using papers from Papers With Code (a repository of Machine Learning papers and their associated code). We complement this body of work by examining world-wide dependencies at the city-level. Scientists’ mobility across institutions has also been studied extensively in the literature^56–62^. Most of the studies have focused on the incentives of scientists from all disciplines to relocate to different countries^63–66^. Some studies have also examined migration between cities, but their focus and goals are different from ours. In particular, Dyachenko^67^ studied the migration of physicists across Russian and American cities. Verginer and Riccaboni^68^ compared global cities to peripheral ones in their ability to attract highly prolific scientists. The same authors explored the mobility network of scientists across cities^69^ focusing on the role of country borders and research output. However, they do not distinguish between Eastern and Western cities, nor consider any particular discipline, AI or otherwise. Moreover, they ranked cities based on Eigenvector centrality, which is different from the novel centrality measure we propose to quantify a city’s role in bridging East and West. Cross-border collaborations have also been considered in the literature^70^, e.g., to study how they are influenced by geographic distance^71,72^ or how they affect national research efforts^73^. Our contribution to this last strand of literature is that we focus on AI citations while exploring the role that different cities play in bridging the East and West. Moreover, using a gravity model, we quantify the importance of size—in terms of papers and number of scientists—on the influence a city exerts on another.

**Figure 1:**
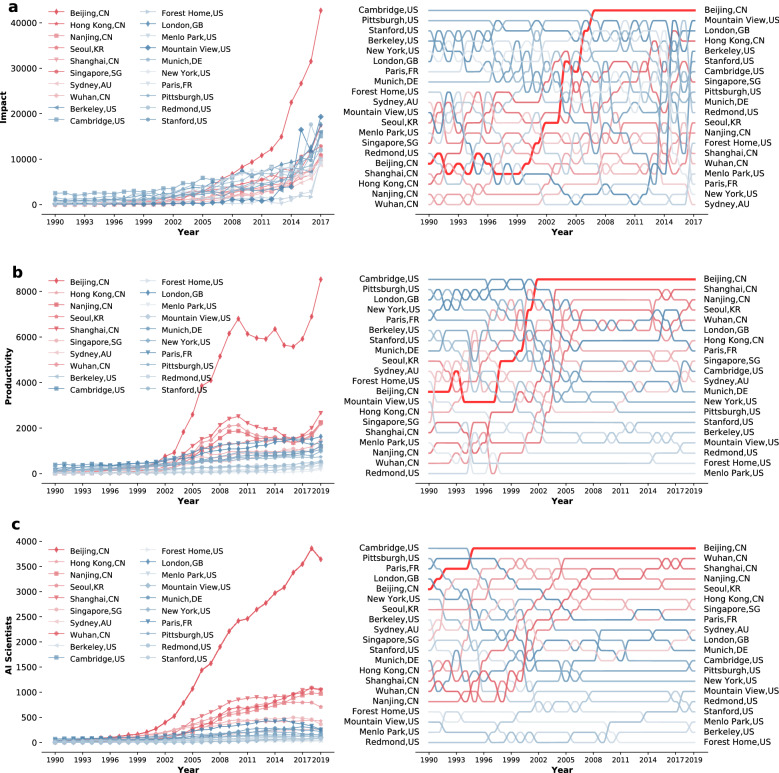
Comparing cities in terms of AI impact, AI productivity, and number of AI Scientists. All subfigures consider the same 20 cities, which were the most impactful in AI in 2017. Cities in the U.S. or Europe are classified as “Western” and colored in blue, while the remaining cities are classified as “Eastern” and colored in red. (**a**) Left panel: AI impact from 1990 to 2017, where greater color intensity indicates greater impact; Right panel: Similar to the left panel, but depicts the relative, rather than the absolute, difference between cities each year. (**b**) Left panel: AI productivity from 1990 to 2019, where greater color intensity indicates greater productivity; Right panel: Similar to the left panel, but depicts the relative, rather than the absolute, difference between cities each year. (**c**) Left panel: Number of AI Scientists from 1990 to 2019, where greater color intensity indicates greater number of AI Scientists; Right panel: Similar to the left panel, but depicts the relative, rather than the absolute, difference between cities each year.

## Results

In the Science of Science literature, several studies use the Microsoft Academic Graph (MAG) dataset^50,74–80^ while several others use the Web of Science (WoS) dataset^81–87^, and some studies even use both datasets^88^. We chose MAG since it provides the strongest emphasis on comprehensiveness^89^. We downloaded the Microsoft Academic Graph dataset^90^ on September 12th, 2020. This dataset includes records of scientific papers specifying the publication date, the publication venue, the publication discipline, the authors’ names, the authors’ affiliations, and the network of citations. Additionally, the dataset specifies the publication type, which includes books, book chapters, conference papers, journal papers, theses, patents, datasets, and repositories. In our study, we only consider publications between 1990 and 2019 (inclusive) whose discipline is specified as AI according to MAG, and whose type is specified as either a conference or a journal paper. Following the convention that the last author is typically the principal investigator and “the head of the lab that hosted most of the research”^91^, throughout our study, the city in which the last author’s affiliation resides is considered to be the city from which the paper has originated. To this end, for any given affiliation, the corresponding city was identified using the Google Map Geocoding API^92^. This yielded a dataset of 2.2 million AI papers that are classified into cities. To quantify the scientific output of each city, we used three outcome measures. The first is *impact*—the number of citations that papers from the city have accumulated within the first two years post publication. Compared to other studies that consider five years^74^ or ten years^93^ post publication, our measure allows us to analyze the impact of more recent papers, which is particularly important when studying a rapidly growing field such as AI. Our second outcome measure is *productivity*—the number of AI papers that the city has produced each year. The third outcome measure is the number of *AI scientists* affiliated to an institution located in the city under consideration.

Figure 1a–c show, respectively, how the impact, productivity, and number of AI scientists have changed over time for the 20 cities that were most impactful in AI in 2017. Throughout the article, we refer to these as the “top 20 cities.” We classify the cities in the U.S. or Europe as “Western,” and classify the remaining cities as “Eastern.” The right-hand side of each subfigure shows how the cities’ ranking has changed over time. Unlike productivity and number of AI scientists, which are depicted up to 2019, impact is only depicted up to 2017 to allow for citations to accumulate for two whole years post publication. Looking at Fig. 1, one can spot Beijing as a clear outlier; it is not only the most impactful city since 2007, but also the most productive since 2002, and the one that has accommodated the largest number of AI scientists for the past two decades. The difference between Beijing and other cities grew to a remarkable level in recent years. In 2017, for instance, Beijing received twice as many citations as the second most impactful city (Mountain View in California, U.S.). In 2019, Beijing produced three times as many papers as the second most productive city (Shanghai) and housed three times as many AI scientists as the city that followed in the ranking (Wuhan).

Supplementary Fig. 1 is similar to Fig. 1a, except that citations are counted five years post publication instead of two. As shown in this figure, the overall trend persists, with Beijing still ranked at the top. Supplementary Fig. 2 shows how much of the global AI research activity is controlled by the top 20 cities. In terms of impact, these cities continued to attract about 20% of global AI citations annually between 1990 and 2013; after this period, the top cities’ share of global AI impact started increasing steadily until it exceeded 30% in 2017, meaning that one in every three citations worldwide go to a paper produced by these cities. In terms of productivity and impact, the top cities produce 18% to 20% of papers worldwide, and house about 20% to 22% of the global AI workforce. Collectively, these results demonstrate the significant role that the top 20 cities play in the rapid development of AI. Supplementary Fig. 3 considers an alternative outcome measure, proposed by Wu et al.^94^. Intuitively, this measure quantifies the degree to which a paper introduces something new that eclipses attention from the previous work upon which it was built, leading other scholars to cite it without citing its references. This measure ranges between 1 and -1; papers whose score is close to 1 are considered “disruptive,” whereas those with a score close to -1 are considered “developmental.” Supplementary Fig. 3a depicts the annual ranking of cities based on the number of papers they produce that are among the 10% most disruptive AI papers each year. As can be seen, Beijing has the highest ranking since 2005. Supplementary Fig. 3b depicts the same plot but for development instead of disruption. Again, Beijing is ranked at the top, and this has been the case since 2003. Finally, Supplementary Fig. 4 compares East to West in terms of publication venues. To this end, for each of the top 20 AI venues^95^, we counted the annual number of Eastern and Western papers published therein; see Supplementary Fig. 4. As shown in this figure, there seems to be a divide between East and West, especially in the last few years, with certain venues being predominantly targeted by the East, and others being mostly targeted by the West.

**Figure 2:**
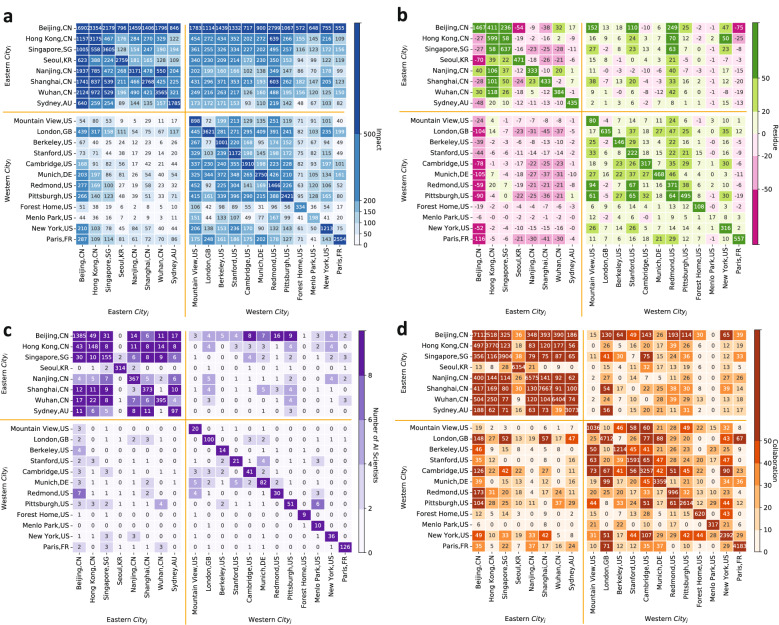
Pair-wise analysis of cities in terms of AI impact, AI over-impact, AI scientists’ migration, and AI scientists’ collaboration. All subfigures consider the same 20 cities, which were the most impactful in AI in 2017. (**a**) The value and color in each cell represent the number of citations from the row city, *i*, to the column city, *j*, in the five periods between 2013 and 2017. To compute this number, for every year *y* in [2013, 2017], and every paper *p* coming from city *j* in year *y*, we consider the citations that *p* received from papers in city *i* during the years *y*, $y+1$, and $y+2$. (**b**) Similar to (**a**) but after subtracting from each cell the expected number of citations according to our baseline model in which citations are random. (**c**) The cells that fall along the diagonal specify the number of scientists whose location in 2015 coincides with their location in 2019; every other cell specifies the number of AI scientists who relocated from the row city to the column city at any point in time during the five years between 2015 and 2019. (**d**) The value and color in each cell represent the number of collaborations from the row city, *i*, to the column city, *j*, in the 5-year period between 2015 and 2019, which is calculated as the number of papers in which the last author is in *i* and at least one coauthor is in *j*.

Figure 2a shows each of the top 20 cities’ impact over other cities during the five years between 2013 and 2017. The rows and columns are ordered such that Eastern cities are grouped together, and Western cities are grouped together. The value and color in each cell correspond to the number of citations from the row city to the column city (colors are binned to improve the visualization; an alternative coloring scheme is provided in Supplementary Fig. 5). It should be noted that impact flows in the opposite direction of citations. For example, a city *j* impacts another city *i* when *i* cites *j*. Thus, in this heatmap, each cell reflects the impact of the column city over the row city, not the other way around. Figure 2a reveals three salient patterns. First, Eastern cities feature little impact on Western cities (see how the color intensity in the bottom-left quadrant is much lower than that of the remaining quadrants). Among Eastern cities, however, Beijing shows the greatest aggregate impact on Western cities; an aggregate impact comparable to that of Berkeley or Cambridge U.S. (without counting the impact that each of the latter cities exerts on itself). Second, Beijing cites all other top cities massively (notice the color intensity of the first row, which is higher than that of any other row). Excluding itself, about 45% of Beijing’s citations are to Eastern cities, and 55% are to Western ones, suggesting that the city’s research builds on knowledge produced across the globe. Third, each city cites itself heavily (see how the color intensity is exceptionally high along the diagonal). This “home bias” is interesting given the fact that communication technologies should, in principle, render distance ineffectual. Our results on AI are consistent with those that document home bias in other fields^96–99^. Analyzing the top 50 cities instead of the top 20 reaffirms the three patterns just described; see Supplementary Fig. 6.

The impact network corresponding to Fig. 2a compares the citations received by different cities in absolute terms, without accounting for differences in productivity. For instance, when comparing Beijing to Mountain View in terms of their impact on Hong Kong, Fig. 2a does not reveal that Beijing produces ten times as many papers as Mountain View. Motivated by this observation, we develop a measure of impact that accounts for the differences in productivity between cities. To this end, we use a simple baseline model in which citations are random. Under this model, the probability of citing a given city is proportional to its productivity. If *n* denotes the number of papers produced globally, $n_j$ denotes the number of papers produced by city *j*, and $m_i$ denotes the number of papers cited by city *i*, then the expected impact of *j* on *i* under our baseline model is $(n_j/n)\times m_i$. This theoretical benchmark is not meant to account for all the variables that determine a scientist’s decision to cite a given paper; its goal is to provide us with a metric suitable to assess the citation preferences of different cities. Take Beijing, for example, which produced about 5% of AI papers worldwide in 2017. For any given city *i*, if more than 5% of the citations made by *i* are to Beijing, it suggests that *i* has a preference for Beijing, in which case we say that Beijing over-impacts *i*. In contrast, if fewer than 5% of *i*’s citations are to Beijing, we say that Beijing under-impacts *i*. More generally, deviations from this benchmark serve as a coarse yet intuitive measure of cities’ preferences. For more details on how the expected impact is calculated, see the Supplementary Note 1.

Figure 2b highlights the cities that over-impact (green) and under-impact (pink) other cities. For any pair of row city *i* and column city *j*, the corresponding cell represents the difference between the number of times *i* cited *j* and the number of times *i* would be expected to cite *j* if citations were determined based on the baseline model. When interpreting this heatmap, recall that impact and citations flow in opposite directions, implying that a pink color indicates that the column city under-impacts the row city. The outcome of this analysis reinforces two of the aforementioned patterns observed in Fig. 2a: Eastern cities under-impact Western ones (see how the bottom-left quadrant has mostly negative values), and all cities over-impact themselves (notice the dark green color along the diagonal). Figure 2b reveals additional patterns: Beijing under-impacts all other top cities (except Wuhan and Nanjing; see the leftmost column), and several top cities under-impact Beijing (most notably Seoul, Shanghai, and Paris; see the upper row). Moreover, many Eastern cities under-impact other Eastern cities; 32 out of the 64 cells in the top-left quadrant contain negative values. The two cities that stand out in this quadrant are Hong Kong and Singapore, which over-impact all top Eastern cities. Globally, only two cities over-impact all other top 20 cities, Mountain View and Redmond, both on the West Coast of the U.S. Finally, the figure reveals the dependency of Western cities on other Western cities; the bottom-right quadrant features mostly positive values. Similar patterns emerge when considering the top 50 cities; see Supplementary Fig. 7.

## Other channels of influence: migration and collaboration

Impact is an important metric to quantify global influence, but it is not the only one. Knowledge diffuses not only through papers but also through people, especially the part of knowledge that cannot be codified. To study this aspect, we identified AI scientists as those who published at least three papers, and at least half of their publications fall under the field of AI according to the Microsoft Academic Graph dataset. Figure 2c shows the number of AI scientists who relocated from the row city to the column city at any point in time during the five years between 2015 and 2019. The diagonal counts the scientists whose location in 2015 coincides with their location in 2019. Out of the four quadrants, the top-left one stands out as the one with the largest values, indicating that the greatest migration of AI scientists occurs between Eastern cities. In this quadrant, Seoul is the only city that neither attracts AI scientists from, nor supplies AI scientists to, other Eastern cities significantly. Figure 2c also reveals that, perhaps unsurprisingly, the vast majority of AI scientists who were in any given city in 2015 remained in that city in 2019 (notice the high color intensity along the diagonal). Once again, Beijing stands out as the one attracting the largest number of scientists from, and supplying the largest number of scientists to, other cities. Supplementary Fig. 8 shows similar migration patterns when considering the top 50 cities.

As an alternative to diffusion from one city to another, knowledge can be created by inter-city teams through collaborations. In Fig. 2d, each cell specifies the number of collaborations from the row city *i* to the column city *j* in the five years between 2015 and 2019; this is calculated as the number of papers in which the last author is in *i* and at least one coauthor is in *j*. As with impact and migration, collaboration between Eastern cities is remarkable (top-left quadrant). Western cities also tend to collaborate frequently with one another (bottom-right quadrant). However, we observe relatively fewer collaborations between the East and the West than within the East or the West (notice the relatively small values in the top-right and bottom-left quadrants). Beijing emerges as a hub, not only for Eastern cities but also for a few Western ones, especially Redmond, Cambridge U.S., London, and Pittsburgh. Considering the top 50 cities instead of the top 20 reveals similar patterns; see Supplementary Fig. 9.

## Beijing’s central role

**Figure 3:**
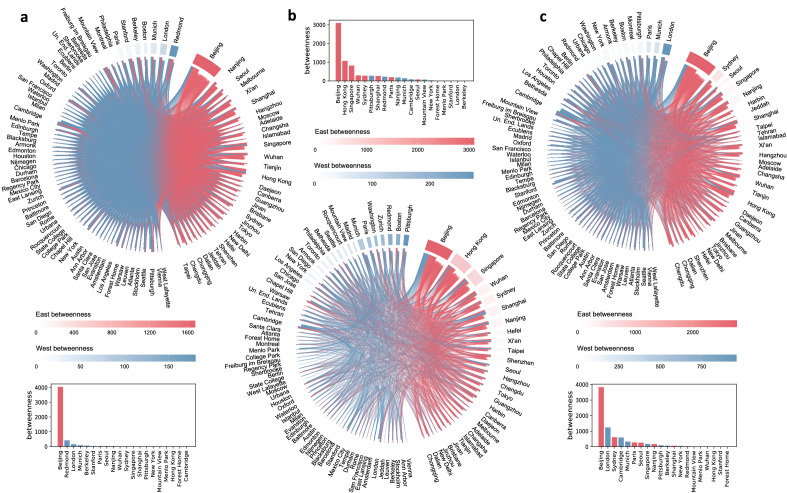
Betweenness centrality of different cities in the networks of impact, migration, and collaboration. Each chord diagram represents a network in which nodes correspond to cities, and colors indicate whether the city is in the East (red) or the West (blue). For each city, the size of the corresponding arc (the portion of the outer ring) reflects the number of edges adjacent to the city, while the arc’s color intensity reflects the city’s betweenness. Cities are grouped into Eastern (right) and Western (left), and are sorted within each group according to betweenness. As for edges in the inner circle, the color indicates whether the edge points to a city in the East (red) or the West (blue), while the thickness indicates the edge’s weight (the interpretation of which differs from one network to another). The bar plots represent the betweenness of the most impactful 20 cities. (**a**) Citation network, where the weight of the edge from city *i* to city *j* represents the number of times an AI paper from *i* cited an AI paper from *j* between 2013 and 2017. (**b**) Migration network, where the weight of the edge from city *i* to city *j* represents the number of AI scientists who relocated from *i* to *j* between 2015 and 2019. (**c**) Collaboration network, where the weight of the edge from city *i* to city *j* represents the number of AI papers from *i* that involved coauthors from *j* between 2015 and 2019.

So far, our analysis of impact, migration, and collaboration suggests that Beijing could be playing a key role in connecting the East and the West. To explore this possibility, we turn to betweenness centrality^100,101^. Roughly speaking, this measure quantifies the importance of any given node in a graph based on the number of shortest paths that go through it. Intuitively, a node with high betweenness centrality can be thought of as a bridge that connects different parts of the network. Since we are particularly interested in quantifying the role that Beijing—and every other city—-plays in connecting the East and the West, we use a modified version of betweenness centrality that only considers paths where the source node falls in the East and the destination node falls in the West, or vice versa; see Methods for a formal definition. Using this measure, we analyze the networks of impact, migration, and collaboration among the top 100 cities, i.e., the 100 cities that were most impactful in AI in 2017. These are identical to the networks corresponding to Fig. 2a,c,d, except that the nodes now represent the top 100, rather than top 20, cities. Note that we do not consider the network of over-impact, i.e., the network corresponding to Fig. 2b, since it has negative weights, making it incompatible with betweenness centrality (Methods). The results of our betweenness-based analysis are depicted in Fig. 3. In all three networks, Beijing has by far the highest betweenness centrality. Let us take a closer look at each network. Starting with impact (Fig. 3a), Beijing’s betweenness is four times greater than the sum of the betweenness of all other cities combined. This finding highlights the vital role that Beijing plays in transitioning knowledge between the East and the West. Intuitively, when a paper *w* in the West cites a paper *b* in Beijing, which in turn cites a paper *e* in the East, the paper *b* can be thought of as a bridge through which the knowledge in *e* is transitioned to *w*. Moving on to the migration network (Fig. 3b), Beijing’s betweenness is three times greater than the city with the second-highest betweenness, suggesting that Beijing acts as the hub through which scientists in the East can reach the West and vice versa. Finally, in terms of collaboration (Fig. 3c), Beijing’s betweenness is greater than the sum of the betweenness of all remaining cities. This can be interpreted as Beijing being the city where Eastern and Western AI scientists most frequently share a coauthor, effectively connecting both parts of the world.

Our findings underline Beijing’s central role in global AI research. As a robustness check, instead of Betweenness centrality, we quantified the roles of different cities using alternative measures, borrowed from the social network analysis toolkit. More specifically, these measures are: (i) degree centrality; (ii) closeness centrality; (iii) PageRank; (iv) influence measured based on the independent cascade model; (v) influence measured based on the linear threshold model; see Supplementary Note 4 for formal definitions. The results of this analysis can be found in Supplementary Tables 4 to 8. As can be seen, regardless of the measure used, or the network under consideration (be it the citation, migration, or collaboration network), Beijing is ranked higher than any other city. These results emphasize Beijing’s role as a central (if not the most central) city in the global AI landscape.

**Figure 4:**
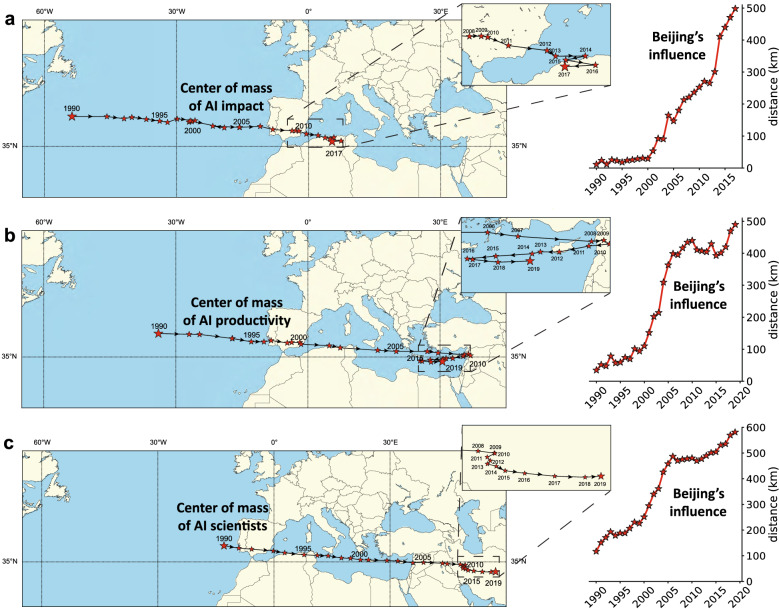
Trajectory of the centers of mass of AI research. (**a**) Left panel: Trajectory of the center of mass of AI impact from 1990 to 2017 (figure generated using the Matplotlib Basemap Toolkit; https://matplotlib.org/basemap/); Right panel: Beijing’s influence, measured each year as the distance (in kilometers) between the center of mass that accounts for all cities (including Beijing) and the one that accounts for all cities except Beijing. (**b**,**c**) are similar to (**a**) but for AI productivity and AI scientists, respectively, from 1990 to 2019.

## Center of mass

To assess the relative strength with which different cities influence the global AI landscape, we conclude our analysis by examining the geographical evolution of AI research over time. We take into consideration three different metrics: impact, productivity, and number of AI scientists. For each metric, and for each year starting from 1990, we compute the *center of mass*—a weighted average of each city’s geographic location (latitude and longitude) weighted by the metric’s value in that city. Intuitively, the center of mass concentrates all the influences that the world’s cities exert on one another into a single geographic point. Figure 4 illustrates its evolution. For all metrics, latitude remained fairly constant over the years—slightly above 35 degrees North—but longitude has been drifting towards the East. As shown in the left panel of Fig. 4a, the center of mass of AI impact was situated in the Atlantic Ocean closer to the U.S. in 1990, but since then, it has moved about 6,500 kilometers eastward. The right panel depicts Beijing’s influence, measured each year as the distance (in kilometers) between the center of mass that accounts for all cities (including Beijing) and the one that accounts for all cities except Beijing. As can be seen, the city’s influence has increased steadily over the years, reaching hundreds of kilometers annually. Moving on to productivity, Fig. 4b shows that the corresponding center of mass moved steadily eastward from 1990 to 2020, landing in Asia. However, the next seven years witnessed a pullback towards the West before the center of mass resumed its initial trajectory. Again, Beijing managed to pull the center of mass hundreds of kilometers eastward in recent years. Finally, Fig. 4c shows that the center of mass of AI scientists has moved more steadily than the previous two centers, crossing about 8,000 kilometers between 1990 and 2019, with an average speed of about 270 kilometers per year, and with Beijing’s influence increasing each year. Taken together, these results indicate an overall drift of global AI research activities towards the East over the past decades, with Beijing playing an influential role in this movement.

## Discussion

By analyzing more than two million AI papers published over decades, we were able to compare cities in terms of their AI research, focusing on the number of papers they produce, the number of citations they receive, and the number of AI scientists they house. Beijing has been the most productive city since 2002, the most impactful since 2007, and the one with the largest AI workforce for the past two decades. To understand the dependencies between Eastern and Western cities, we started off by studying the impact network of the most impactful cities worldwide. We found that (i) all cities cite themselves far more frequently than they cite others; (ii) top Eastern cities exert a relatively small impact on top Western cities; and (iii) Beijing cites other cities the most, regardless of whether their location lies in the East or the West, suggesting that its research builds on knowledge produced across the globe. We compared the observed citation patterns to those expected under a baseline model in which citations occur randomly. This analysis revealed a substantial dependency of Western cities on other Western cities, as they cite each other far more frequently than expected by chance. Next, we analyzed the migration of AI scientists between the top cities from 2013 to 2017. We found that migration mostly takes place from an Eastern city to another Eastern city. When studying the network of collaborations across cities, we found that East–East collaborations are far more frequent than East–West or West–West collaborations. Motivated by these observations, we set out to identify the cities bridging East and West. To this end, we proposed a version of betweenness centrality that only considers the shortest paths starting in the East and ending in the West, or vice versa. Using this measure, we analyzed the impact network, the migration network, and the collaboration network of the most impactful cities in AI worldwide. Beijing’s betweenness is far greater than that of any other city, regardless of the network under consideration, highlighting Beijing’s key role in bridging Eastern and Western cities. Finally, we tracked the center of mass of AI research, which weighs the locations of different cities by their output, be it AI impact, AI productivity, or AI workforce. The three centers of mass have all been drifting towards the East since 1990, with Beijing contributing significantly towards this phenomenon. Taken together, these findings underscore the growing role of the East in general, and Beijing in particular, in shaping the global AI landscape.

Our results suggest that size matters. By comparing Fig. 2a,b we can see that, once we control for the total number of papers of the source city (i.e., the city that produces the paper being cited) and control for the total number of citations of the destination city (i.e., the city that cites the source), Beijing’s impact on Western cities becomes limited. In other words, Beijing influences other cities by virtue of its size. In Supplementary Table 1 we see that the association between size and citations is close to one-to-one on average across all cities (for details, see Supplementary Note 1). Just like size, other variables could explain variation in citations between cities. The simple gravity model that only includes size variables can be extended to a richer gravity model that accounts also for number of AI scientists, distance and cultural-geographic elements^102,103^. Table 1 shows the estimates for the main size variables in drawing Fig. 2b—productivity at source and total citations of destination—but now including these other variables (“frictions”) as controls. The estimates associated with the frictions and the details of the procedure are described in Supplementary Note 2. In Table 1 specification 1, we include the baseline frictionless model used to draw Fig. 2b. The coefficients on source city’s productivity and on destination city’s total citations remain qualitatively the same as we add: number of AI scientists at source and destination (specification 2), distance (specification 3), and cultural-geographic factors such as language and country, among others (specification 4). These results reinforce the idea that even the simple gravity model used earlier (i.e., specification 1) provides a good account of inter-city impact. The estimates associated with each friction used as control are in Supplementary Table 2. It is worth noting that some controls are strongly associated with impact. Distance, in particular the same-city indicator, stands out even after controlling for country and language. Number of AI scientists at the source seems to have some explanatory power although its correlation with citations is not robust to specification. The same goes for AI scientists at destination. But number of scientists does seem to matter when studying migration. Drawing on the same idea that migration between cities depends on the size of those cities (now measured by the number of AI scientists) and also frictions (^102^ p. 32), we can fit a between-city migration model using Pseudo Poisson Maximum Likelihood. We present the full set of results in Supplementary Table 3. An important finding is that total number of AI scientist at destination is strongly and significantly associated with migration between cities. And so is total AI scientists at the source. Both results suggest that cities housing many AI scientists are attractive destinations for other AI scientists, but larger cities also supply more scientists to the world, as one would expect. As such, these results are consistent with the idea that size, now in terms of human capital, matters for migration decisions. For details, see Supplementary Note 3.

**Table 1:** Gravity model: citations.

Dependent variable:	Citations to source city *j*’s papers from city *i* (Impact of *j* on *i*)							
	(1)		(2)		(3)		(4)	
Source’s productivity ($n_j$)	1.145***	(0.029)	1.340***	(0.085)	1.324***	(0.085)	1.250***	(0.081)
Total citations by city *i* ($m_i$)	1.000***	(0.017)	1.002***	(0.037)	0.951***	(0.028)	0.894***	(0.024)
Constant	− 12.509***	(0.110)	− 12.814***	(0.224)	− 12.173***	(0.306)	− 13.014***	(0.315)
Controls								
Number of AI Scientists	No		Yes		Yes		Yes	
Distance	No		No		Yes		Yes	
Cultural & Geographic	No		No		No		Yes	
N	6119444		3084448		3084448		3063400	
Pseudo R-sq	0.568		0.559		0.630		0.654	
Ho: coefficient $n_j$ = 1 *p*-val.	<0.001		<0.001		<0.001		0.002	
Ho: coefficient $m_i$ = 1 *p*-val.	0.99		0.95		0.08		<0.001	

The table shows the estimation of the Poisson Pseudo-Maximum Likelihood estimation of the gravity model of citations. Column (1) shows the point estimates of the baseline frictionless model as benchmark. The remaining columns present the estimates of models with frictions as controls added sequentially. The number of observations decreases after including controls because of missing observations on the number of scientists, and cultural-geographic variables. The last two rows show the *p*-values associated with a one degree-of-freedom chi-squared test where the null hypothesis is the corresponding coefficient being equal to 1. The Table with all the estimates is in Supplementary Table 2. Standard errors are in parentheses to the right of each coefficient. Stars next to each estimate denote significance at conventional levels (*p-value < 0.05, **p-value < 0.01, ***p-value < 0.001).

Nations are racing to dominate the field of AI given its disruptive and transformative potential^1^. Our study has shown that dependencies matter, so competition may not be the right approach to understand the evolution of AI. China’s capital already plays a critical role in AI research not only as a hub of knowledge creation but also as a bridge between East and West in terms of citations, collaborations, and scientists’ migrations. More often than any other city, Beijing seems to pick scholarly knowledge from one side of the globe, build on it, and then introduce it to the other side, acting as a gateway through which knowledge flows in both directions. Whereas researchers from prominent cities on the West Coast of the U.S. largely collaborate with scientists in other Western cities, Beijing-based researchers collaborate with scholars located in both East and West, possibly tapping into tacit knowledge and resources available to scientists across the globe. Also, more often than any other city, Beijing sees AI scientists passing through from one side of the world to the other; those scientists probably leave traces of their expertise and technical know-how along the way. Taken together, these findings highlight Beijing’s central role in global Artificial Intelligence research.

A noteworthy limitation of our study is that we restrict our attention to AI papers while disregarding other forms of research. This choice was based on the fact that bibliometrics provide all the information required for our investigation, such as, e.g., the number of citations received (which allows us to quantify impact using an established measure) as well as the identities and affiliations of each author (which allow us to track the movement of scientists across cities). Having said that, bibliometrics alone do not provide a complete picture of global AI research. Other aspects include the amount of funding spent in, and the number of patents produced by, different cities. Future work may consider the number of big-tech companies and start-up companies that reside in different cities, as well as the growth and revenue of these companies. Another avenue for future work is to analyze the subfields of AI, to determine which areas of research, if any, attract attention from the East but not the West, or vice versa. In terms of policy implications, there is room for initiatives that promote intra- and inter-national migration of and collaboration among researchers. We observe a divide between East and West that so far seems to be bridged by Beijing, but other cities may also provide such a bridge. If the goal is to advance humankind's AI knowledge, policy makers could also foster cities such as Hong Kong or Redmond U.S. as hubs by attracting knowledge and researchers from East and West, thereby diversifying their pool of talents and skills. Our findings may also inform AI scientists’ migration decisions, bearing in mind that the cities serving as hubs of AI knowledge and talent are likely to drive AI research in the coming years. Overall, these findings suggest that efforts from funders, research institutions, and scientists that aim at bridging the East–West divide could pay off. Building more bridges across the world could start by promoting incentives to share research more broadly across venues or to create more inclusive publication venues.

## Methods

Given a directed weighted network, let *V* denote the set of vertices (i.e., nodes) in the network. Then, given two nodes, $x,y\in V$, a path from *x* to *y* is said to be a shortest path if it minimizes the sum of the edge weights along the path. Let us now define betweenness centrality.

### Definition 1

For any two nodes, $x,y\in V$, let $\sigma _{xy}$ be the number of shortest paths from *x* to *y*, and let $\sigma _{xy}(v)$ be the number of shortest paths from *x* to *y* that go through *v*. Then, betweenness centrality is a function $b: V \rightarrow \mathbb {R}$ defined for every $v\in V$ as:

1 $$\begin{aligned} b(v) = \sum _{x,y \in V\setminus \{v\} : \sigma _{xy} > 0} \frac{\sigma _{xy}(v)}{\sigma _{xy}} \end{aligned}$$

Note that Definition 1 requires $\sigma _{xy}$ to be greater than zero. The reason behind this requirement is to avoid division by zero whenever the network under consideration is not strongly connected. To understand why this is the case, suppose a node $y^*$ is not reachable from a certain other node $x^*$. In this case, to compute the betweenness of a node *v*, we would have to count the number of shortest paths that go through *v* out of all shortest paths from $x^*$ to $y^*$, which would lead to a division by zero since there are no shortest paths from $x^*$ to $y^*$. One way to address this problem is to restrict the analysis to pairs of nodes that have a shortest path between them, i.e., by excluding pairs similar to the aforementioned $(x^*,y^*)$ from the analysis. This exclusion is done by requiring $\sigma _{xy}$ to be greater than zero, as in Definition 1.

Traditionally, betweenness centrality is applied in scenarios where edge weight represents distance, and where the flow of information between any two nodes follows a shortest path. As such, the greater the number of shortest paths that go through a given node, the greater the control that this node has over the network, since more information passes through it. However, the situation is different in the networks considered in our analysis, i.e., impact, migration, and collaboration. In each of these networks, a node is considered more central if it falls on paths that maximize, rather than minimize, the sum of edge weights. As such, to stay faithful to the intuitive interpretation of betweenness centrality, we replace each edge weight with its reciprocal before computing the centrality of each city. Importantly, since we are particularly interested in quantifying the role that different cities have in connecting the East and the West, we introduced a modified version of betweenness that only considers paths of which the source node falls in the East and the destination node falls in the West, or vice versa. Let us formally define this modified version, which we call East–West betweenness. In particular, the nodes in our setting correspond to cities that are classified into East and West, depending on their geographic location. Let *E* and *W* be the subsets of *V* consisting of the nodes that correspond to Eastern and Western cities, respectively, implying that $E \cup W = V$ and $E\cap W =\emptyset$. Then, East–West betweenness is defined as follows:

### Definition 2

For any two nodes, $x,y\in V$, let $\sigma _{xy}$ be the number of shortest paths from *x* to *y*, and let $\sigma _{xy}(v)$ be the number of shortest paths from *x* to *y* that go through *v*. Then, the East–West betweenness centrality is a function $b: V \rightarrow \mathbb {R}$ defined for every $v\in V$ as:

2 $$\begin{aligned} b(v) = \sum _{e \in E\setminus \{v\}, w \in W\setminus \{v\} : \sigma _{ew}> 0} \frac{\sigma _{ew}(v)}{\sigma _{ew}}\ \ + \sum _{e \in E\setminus \{v\}, w \in W\setminus \{v\} : \sigma _{we} > 0} \frac{\sigma _{we}(v)}{\sigma _{we}} \end{aligned}$$

Finally, let us discuss the reason behind excluding the network of over-impact (i.e., the network corresponding to Fig. 2b) from our betweenness-based analysis. As mentioned earlier, betweenness centrality is traditionally applied in scenarios where edge weight represents distance. Since there is no such thing as negative distance, betweenness is not applicable when some of the edge weights are negative, which is the case with the over-impact network. As an alternative, we computed betweenness on a network of normalized impact, where edge weights are computed as per Equation (2) in^50^. That is, the weight of the edge from city *i* to city *j* is:

$$\begin{aligned} w_{ij} = \frac{\text {(share of citations to }j'\text {s AI papers that came from }i'\text {s AI papers)}}{(j'\text {s share of global AI papers)}} \end{aligned}$$

With this normalization, if *j* produced just a single paper, and this paper got cited by *i*, then the edge (*i*, *j*) will be assigned the maximum possible weight. Such extreme edges will greatly influence the East–West betweenness of cities that are adjacent to them, making the measure highly sensitive to outliers. To appreciate the difference between the normalized and non-normalized networks, consider a stylized example with three cities, $\{x_1, x_2, x_3\}$, such that $x_1$ produces a single paper that gets cited by $x_2$, which in turn produces a single paper that gets cited by $x_3$. Furthermore, consider three other cities, $\{y_1,y_2,y_3\}$, such that $y_1$ produces 10,000 papers, 9,000 of which get cited by $y_2$, which in turn produces 10,000 papers, 9,000 of which get cited by $y_3$. In the normalized network, all other things being equal, $x_2$ would be more central than $y_2$ because of the greater weights of its edges. On the other hand, in the non-normalized network, $y_2$ would be more central than $x_2$ because of the far greater number of citations that flow through it. In our study, we focus on the non-normalized version, since we are interested in identifying the cities through which the largest number of citations flow between East and West. Nevertheless, to provide a comprehensive analysis, we computed the East–West betweenness in the normalized network; see Supplementary Fig. 10. Indeed, smaller cities are, on average, ranked much higher than larger ones according to this analysis. For instance, the two highest ranked Eastern cities are Jeddah and Islamabad, which are very small in the grand scheme of AI research: Jeddah is ranked 339th according to productivity and 48th according to impact, while Islamabad is ranked 166th according to productivity and 95th according to impact.

## Supplementary Information

Supplementary Information.

## Data Availability

All data generated or analysed during this study can be downloaded from the Microsoft Academic Graph^90^ website: https://www.microsoft.com/en-us/research/project/microsoft-academic-graph/.
