# Supplementary material for: Beijing’s central role in global artificial intelligence research
Source: Sci Rep. 2022 Dec 12;12:21461. doi: 10.1038/s41598-022-25714-0 (PMC9744801; doi:10.1038/s41598-022-25714-0)
Supplement: Supplementary file 1 — Supplementary Information. [file 41598_2022_25714_MOESM1_ESM.pdf]

Supplementary Materials for

Beijing's Central Role in Global Artificial Intelligence Research

Bedoor AlShebli , Enshu Cheng , Marcin Waniek , Ramesh Jagannathan , Pablo Hernández-Lagos*, and Talal Rahwan*

*Joint corresponding authors, ordered alphabetically: pablo.hernandezlagos@yu.edu; talal.rahwan@nyu.edu

Supplementary Note 1: Actual and Expected Impact

In the context of AI research, cities differ in various ways. Beijing, for example, received over 42,000 citations in 2017, which is about twice as much as those received by Mountain View, California. However, in that year, Beijing produced five times as many papers as Mountain View. Thus, although Beijing was more impactful than Mountain View in absolute terms, one could argue that it was less impactful in relative terms, considering the differences in productivity between the two cities. In this section, we propose a benchmark for impact that accounts for productivity using standard techniques drawn from the field of international trade in economics.

The benchmark builds upon a model of expected impact based on the assumption of random citations. As described in the paper, if we denote by n the number of papers produced globally, n_i the number of papers produced by city i, and m_j the number of papers cited by city j, then the expected impact of i on j under the random citation baseline model is $(n_i/n) \times m_j$. This is the basic version of a frictionless gravity model widely studied in the international trade literature [1, 2, 3]. We follow a standard procedure from that literature to estimate the expected impact for each city pair in the sample. According to this procedure, the impact of city i on city j, which we label m_{ij}, is given by

$$m_{ij} = n^{-1} n_i^{\alpha} m_j^{\beta} \mu_{ij} \tag{1}$$

where μ_{ij} is an error term statistically independent of n_i and m_j with $E[\mu_{ij}|n_i, m_j] = 1$, while α and β capture the relative importance of the productivity of i and the number of papers cited by j, respectively. The expected impact, conditional on n_i and m_j, is equal to $E[m_{ij}|n_i, m_j] = n^{-1} n_i^{\alpha} m_j^{\beta}$. We estimate α and β to determine the expected impact empirically. In particular, if $\alpha = \beta = 1$ then the expected impact described by Equation (1) coincides with the random citation baseline model.

We estimate Equation (1) using standard techniques developed in the international trade literature. We follow the approach in [4] and compute the Poisson pseudo-maximum likelihood estimator (PPML) to calculate the expected impact. Poisson estimators are flexible enough to estimate Equation (1) directly, without the need to linearize it, thereby retaining city pairs for which the citation values are zero. Furthermore, Poisson estimators do not require the homoskedasticity assumption. Supplementary Table 1 presents the estimation of the exponents in Equation (1). Each column shows the results for a year between 2013 and 2017. As can be seen, although we reject the null hypothesis that $\hat{\alpha} = 1$ at conventional levels in each specification, the estimated coefficients $\hat{\alpha}$ and $\hat{\beta}$ are very close to 1 across columns. In year 2017 for example, using $N = 6,119,444$ city-pairs yields a coefficient $\hat{\alpha} = 1.145$ (s.e. 0.029) and $\hat{\beta} = 1.000$ (s.e. 0.017); a constant coefficient -12.509 (s.e. 0.110), which implies a baseline citation between i and j of $e^{-12.509} \approx 0$ and a pseudo-R^2 of 0.568. The precision with which the coefficients are estimated suggests that the random citation model provides a good approximation on average, but there may be other variables that might capture part of the unexplained variation. In any case, these empirical results align with the random citation model, albeit not perfectly.

We compute the difference between actual impact m_{ij} and expected impact $\hat{m}_{ij} = n^{-1} n_i^{\hat{\alpha}} m_j^{\hat{\beta}}$ for each year from 2013 to 2017 using the estimates in Supplementary Table 1. In Figure 2b, as well Supplementary Figure 7, each cell corresponds to the sum of the difference $(m_{ij} - \hat{m}_{ij})$ for each city-pair (i, j) across the five years from 2013 to 2017. Intuitively, this represents the difference between actual and expected impact during those five years.

Supplementary Table 1: Estimation of expected impact.

	(1)	(2)	(3)	(4)	(5)
Year	2013	2014	2015	2016	2017
Productivity (α)	1.054***	1.080***	1.117***	1.148***	1.145***
	(0.031)	(0.024)	(0.024)	(0.028)	(0.029)
Total citations (β)	1.000***	1.000***	1.000***	1.000***	1.000***
	(0.018)	(0.021)	(0.019)	(0.019)	(0.017)
Constant	-11.973***	-12.141***	-12.344***	-12.511***	-12.509***
	(0.108)	(0.083)	(0.105)	(0.106)	(0.110)
N	5,692,960	5,925,577	6,035,508	6,202,547	6,119,444
pseudo-R^2	0.297	0.409	0.434	0.501	0.568
Ho: coeff. of n_i = 1, p-val:	0.078	0.001	<0.001	<0.001	<0.001
Ho: coeff. of m_j = 1, p-val.:	0.999	0.999	0.999	0.999	0.999

The table shows the estimation of the Poisson Pseudo-Maximum Likelihood estimation of the gravity model of citations estimated each year separately. The last two rows show the p-values associated with a one degree-of-freedom chi-squared test where the null hypothesis is the corresponding coefficient being equal to 1. Standard errors are in parentheses to the right of each coefficient. Stars denote significance at conventional levels ($*$ p-value$<$0.05, $**$ p-value$<$0.01, $***$ p-value$<$0.001)

Supplementary Note 2: Gravity model of citations with frictions

The model in Equation (1) represents a world without frictions. Frictions can be readily incorporated into the model ([5] p. 17). Commonly used proxies for frictions are: distance, country's contiguity, common language, whether English is the official language of country, previous colonial ties and remoteness (weighted average distance of a city to the world, weighted by number of AI scientists). After including frictions, the model in Equation (1) becomes:

$$m_{ij} = n^{-1} n_j^{\alpha} m_i^{\beta} F_{ij} \mu_{ij} \tag{2}$$

where the additional factor, $F_{ij} = \prod_k (f_{ij}^k)^{\theta_k}$, is a function of the frictions defined. The goal is to estimate α, β and the set of parameters θ_k. The error term μ_{ij} makes the relationship hold with equality and follows the same distributional assumptions described in the previous section. Note that Equation (1) is a particular case of Equation (2) when $\theta_k = 0$ for all k.

We follow [4] and estimate Equation (2) using a Pseudo-Maximum Likelihood (PML) estimator that accounts for zero citations. We divide the additional variables in three groups. The first group comprises total scientists at source and at destination. The second group consists of a series of indicator variables that account for distance, including the same-city indicator. We compute geodesic distance between two cities using their coordinates, downloaded from the Google Geocoding API. The last group includes cultural and geographic variables. We use the CEPII variables [6] widely utilized in the trade literature. These variables are indicators of whether two city's countries: are contiguous, share official common language, shared common language is English, ever had a colonial relationship, colonial relationship extends after 1945, and share a common colonizer. We include two additional geographic variables that measure remoteness of both source and destination. Remoteness is simply the weighted average of the distance to all other cities in the world, with weights being the share of AI scientists of those cities over the total number of AI scientists in the world. Supplementary Table 2 shows the Poisson PML estimates using different specifications. Impact is positively and significantly associated with productivity, regardless of the model and specification. Column (1) shows the basic frictionless model estimation for clarity. This result coincides with column (5) in Supplementary Table 1. Each of the remaining columns adds frictions as controls sequentially. Column (2) includes number of AI scientists. The coefficients accompanying both, productivity and destination total citations, remain qualitatively the same as in the frictionless model (1). However, the estimates for the parameters of both source and destination's total AI scientists are small in magnitude with relatively large standard errors. Column (3) adds distance. Again, the parameters accompanying productivity and destination total citations remain about the same and precisely estimated. Regarding distance, we observe evidence of home-bias in that citations are positively associated with being in the same city. But closeness also seems to matter. Cities within a radius of about 6,000 km are associated with additional citations. The last column adds geographic and cultural controls. The coefficients in the frictionless model remain very close to one, although we reject equality at conventional levels. Regarding controls, same country and common language are the ones that stand out as positive. Colonial ties after 1945, however,

are negatively associated with citations. Remoteness of the source city, that is, how far the city is relative to all other cities, is positively associated. This may indicate that some small centers such as Redmond in Washington State, U.S. elicit a large number of citations.

The main conclusion from the gravity model with frictions is the robustness of the frictionless model. That is, size of the source in terms of productivity and of the destination in terms of total citations made, provide fairly good explanations of the expected number of citations a city receives through a frictionless gravity model. Nevertheless, frictions explain some of the unaccounted variance. Geographic and cultural closeness, for example, are also positively associated with impact.

Supplementary Table 2: Gravity model: citations

Dependent variable:	Citations to source city j's papers from city i (Impact of j on i)							
	(1)		(2)		(3)		(4)	
Source's productivity (n_j)	1.145***	(0.029)	1.340***	(0.085)	1.324***	(0.085)	1.250***	(0.081)
Total papers cited by city i (m_i)	1.000***	(0.017)	1.002***	(0.037)	0.951***	(0.028)	0.894***	(0.024)
Number of AI scientists:								
Total scientists at source city j			-0.179*	(0.071)	-0.282***	(0.069)	-0.210**	(0.066)
Total AI scientists at city i			-0.001	(0.041)	-0.047*	(0.023)	-0.002	(0.016)
Distance:								
Same city					3.592***	(0.283)	3.047***	(0.249)
Distance; 2000km					0.786***	(0.073)	0.587***	(0.072)
4000km					0.393***	(0.093)	0.402***	(0.102)
6000km					0.217**	(0.083)	0.435***	(0.104)
8000km					0.029	(0.059)	0.363***	(0.071)
10000km					0.214*	(0.093)	0.573***	(0.118)
12000km					0.257**	(0.082)	0.542***	(0.096)
14000km					0.123	(0.081)	0.262***	(0.079)
16000km					0.006	(0.071)	0.037	(0.072)
Cultural and geographic variables:								
Same country							1.062***	(0.070)
Contiguous country							0.142	(0.077)
Official common language							0.448***	(0.116)
Official common language as English							-0.152	(0.081)
Colonial relationship							0.059	(0.088)
Colonial relationship after 1945							-0.316***	(0.094)
Common colonizer after 1945							0.297**	(0.104)
Remoteness of source j							0.185***	(0.034)
Remoteness of destination i							-0.001	(0.019)
Constant	-12.509***	(0.110)	-12.814***	(0.224)	-12.173***	(0.306)	-13.014***	(0.315)
N	6119444		3084448		3084448		3063400	
pseudo R-sq.	0.568		0.559		0.630		0.654	
Ho: coefficient n_i = 1 p-val.	<0.001		<0.001		<0.001		0.002	
Ho: coefficient m_j = 1 p-val.	0.99		0.95		0.08		<0.001	

The table shows the estimation of the Poisson Pseudo-Maximum Likelihood estimation of the gravity model of citations. Column (1) shows the point estimates of the baseline frictionless model as benchmark. The remaining columns represent models with frictions as controls added sequentially. The number of observations decreases after including controls because of missing observations on the number of scientists, and cultural-geographic variables. The last two rows show the p-values associated with a one degree-of-freedom chi-squared test where the null hypothesis is the corresponding coefficient being equal to 1. Standard errors are in parentheses to the right of each coefficient. Stars denote significance at conventional levels ($*$ p-value<0.05, $**$ p-value<0.01, $***$ p-value<0.001)

Supplementary Note 3: Gravity model of migration with frictions

Drawing on the previous analysis and the rich literature on gravity models, we can also estimate a model of migration that accounts for size and frictions. Equation 2 can be relabeled to explain migration of AI scientists from one city to another. Now, let m_{ij} denote migration from city j to city i, n_i AI scientists supplied to i from all origins, m_j total AI scientists in j, and n total AI scientists in the world. Using n_i allows us to account for whether migration decisions are associated with the number of scientists at destination. Such decisions might be motivated by agglomeration economies or knowledge spillovers at destination that may enhance the migrant scientist's productivity. The inclusion of m_j accounts for the rather mechanical effect that larger cities should source more scientists to the rest of the world. The migration model is a straightforward relabelling of the citation model above. But note that the number of pairwise observations for scientist migration is smaller than for citations. That is due to the fact that one scientist can write a large number of papers but migrate very few times. Another clarification is that we use productivity of i and total citations made by j as controls to account for production size frictions. Finally, we measure migration in five-year intervals as people's mobility is slower than knowledge diffusion. Thus, we focus on the period 2015 to 2019.

Supplementary Table 3 shows the results of the estimation. Column (1) features the frictionless model. Total AI scientists supplied to i is positively associated with migration to city i, although the value is far from 1. AI labor size at source also seems to matter but the coefficient is estimated less precisely. When adding productivity and impact controls in column (2) the coefficient on total AI scientists supplied to city i doubles and remains significant at conventional levels. However, adding the productivity and impact controls does little to the correlation between AI labor at source and migration. Column (3) and (4) add distance and geographic-cultural controls sequentially. In both specifications, the coefficients on AI labor supplied to city i and AI labor in city j are positive (about 0.5) and precisely estimated. These results suggest that AI scientists migrate more frequently to cities that attract other AI scientists from all over the world. They also suggest that the number of AI scientist in a city is associated with higher chances of finding an AI scientist from that city everywhere else. The fact that frictions make the coefficient on AI labor larger and more precise suggest that frictions matter for migration, perhaps more than for citations.

Supplementary Table 3: Gravity model: Migration

Dependent variable:	AI Scientists' migration to destination city i from city j (Migration from j to i)							
	(1)		(2)		(3)		(4)	
AI scientists supplied to i from all origins	0.238***	(0.031)	0.506***	(0.057)	0.603***	(0.025)	0.566***	(0.026)
Total AI scientists at city j	0.217**	(0.076)	0.231*	(0.091)	0.448***	(0.035)	0.450***	(0.031)
Productivity and citations:								
Destination's productivity			0.427***	(0.127)	-0.008	(0.027)	0.017	(0.028)
Total papers cited by city j			-0.620***	(0.083)	-0.106***	(0.021)	-0.095***	(0.022)
Distance:								
Same city					3.723***	(0.146)	4.033***	(0.184)
Distance; 2000km					0.067	(0.137)	0.121	(0.135)
4000km					-0.104	(0.130)	-0.271*	(0.131)
6000km					0.304	(0.171)	0.144	(0.148)
8000km					-0.074	(0.133)	-0.046	(0.129)
10000km					-0.202	(0.124)	-0.136	(0.124)
12000km					0.169	(0.172)	0.236	(0.170)
14000km					-0.295	(0.164)	-0.279	(0.150)
16000km					0.064	(0.178)	0.061	(0.177)
Cultural and geographic variables:								
Same country							-0.228*	(0.102)
Contiguous country							0.223	(0.130)
Official common language							0.361***	(0.089)
Official common language as English							-0.131***	(0.032)
Colonial relationship							0.242*	(0.113)
Colonial relationship after 1945							0.109	(0.207)
Common colonizer after 1945							0.715***	(0.194)
Remoteness of source							-0.041*	(0.017)
Remoteness of destination							-0.051	(0.029)
Constant	-0.696	(0.452)	-0.090	(0.666)	-3.735***	(0.213)	-3.855***	(0.202)
N	8306		4146		4146		4139	
pseudo R-sq	0.022		0.033		0.972		0.979	
Ho: coefficient AI scientists at $i = 1$ p-val.	<0.001		<0.001		<0.001		<0.001	
Ho: coefficient AI scientists at $j = 1$ p-val.	<0.001		<0.001		<0.001		<0.001	

The table shows the estimation of the Poisson Pseudo-Maximum Likelihood estimation of the gravity model of migrations. Column (1) shows the point estimates of the baseline frictionless model as benchmark. The remaining columns represent models with frictions as controls added sequentially. The last two rows show the p-values associated with a one degree-of-freedom chi-squared test where the null hypothesis is the corresponding coefficient being equal to 1. Standard errors are in parentheses to the right of each coefficient. Stars denote significance at conventional levels ($*$ p-value<0.05, $**$ p-value<0.01, $***$ p-value<0.001)

Supplementary Note 4: Alternative network science measures

In this section, we quantify the importance of each city in the citation network, the collaboration network, and the migration network using various measures borrowed from the social network analysis toolkit. To this end, for any given node $v_i \in V$, let $P(v_i)$ denote the set of all predecessors of v_i (i.e., all nodes that have edges to v_i), let $S(v_i)$ denote the set of all successors of v_i (i.e., all nodes with edges from v_i), let w_{ij} denote the weight of the edge from v_i to v_j, and let d_{ij} denote the distance between v_i and v_j (i.e., the sum of weight reciprocals along the shortest path between the two nodes). With this notation in place, we are ready to formally define three centrality measure:

- Degree centrality [7]—the importance of a node v_i is determined based on the weights of the edges incident with v_i. That is:

$$c_{degr}(v_i) = \sum_{v_j \in P(v_i)} w_{ji} + \sum_{v_j \in S(v_i)} w_{ij}$$

- Closeness centrality [8]—the importance of a node v_i is determined based on the distance from v_i to all other nodes (if there is no path from v_i to v_j we assume $d_{ij} = \infty$). More formally:

$$c_{clos}(v_i) = \sum_{v_j \in C \setminus \{v_i\}} \frac{1}{d_{ij}}$$

- PageRank centrality [9]—the importance of a node v_i is determined based on the impotance of its neighbors. The PageRank centrality is computed by an iterative process, where the centrality of each node v_i in the first round is $c^1_{page}(v_i) = \frac{1}{|V|}$. In a subsequent round, t, the centrality of node v_i is computed as:

$$c^t_{page}(v_i) = \frac{1-\gamma}{|V|} + \gamma \sum_{v_j \in P(v_i)} \frac{w_{ji}}{\sum_{v_k \in S(v_j)} w_{jk}} c^{t-1}_{page}(v_j)$$

  where $\gamma = 0.85$ is the damping factor. We continue the computation until one of the following two conditions is satisfied: (1) the computation lasts for 1,000,000 iterations; (2) the difference in the centrality sum of all nodes between does not exceed 10^{-5} for 1,000 consecutive iterations.

In additional to the above three centrality measures, we also consider two alternative influence measures. Both measures are based on the idea that influence may propagate through a network by "node activation". The basic idea is as that when a certain node is sufficiently influenced by its neighbours, it becomes "active", in which case it starts influencing its "inactive" neighbours. To initiate this influence propagation process, one of the nodes, called *the seed node*, is activated from the start. Assuming that time is discrete, we denote by $I_t \subseteq V$ the set of nodes that are active at round t, implying that I_1 is the set consisting of the seed node. The way influence propagates to inactive nodes depends on the influence model under consideration. In this context, two widely-used models are:

- Independent cascade [10]—in every round $t > 1$, every node $v_i \in V$ that became active in round $t-1$ activates every inactive successor, $v_j \in S(v_i) \setminus I_{t-1}$, with probability:

$$p_{ij} = \frac{qw_{ij}}{w^*},$$

  where $q = 0.25$ is the basic activation probability, and w^* is the maximal edge weight in the network. The process ends when there are no newly active nodes, i.e., when $I_t = I_{t-1}$.

- Linear threshold [11]—every node $v_i \in V$ is assigned a *threshold value* θ_i which is uniformly sampled from the $[0, 1]$ interval. Then, in every round $t > 1$, every inactive node v_i becomes active, i.e., becomes a member of I_t, if the total weight of edges from their active predecessors divided by the total weight of all incoming edges meets or exceeds the threshold, i.e., if:

$$\frac{\sum_{v_j \in P(v_i) \cap I_{t-1}} w_{ji}}{\sum_{v_j \in P(v_i)} w_{ji}} \geq \theta_i$$

  The process ends when there are no newly active nodes, i.e., when $I_t = I_{t-1}$.

In either model, the influence of a node v_i is defined as the expected number of active nodes when starting with v_i as the seed node.

Our results are presented in Tables 4 to 8. As can be seen, regardless of the network under consideration (be it the citation, the collaboration, or the migration network) and regardless of the measure used (be it degree, closeness, PageRank, independent cascade-based influence, or linear threshold-based influence) Beijing is the highest ranked city worldwide.

Supplementary Table 4: City ranking according to **degree centrality** in the citation, migration, and collaboration networks. The color indicates whether the city is east (red) or west (blue).

	Citation network			Migration network			Collaboration network	
1)	Beijing	396.525	1)	Beijing	3.298	1)	Beijing	71.576
2)	Hong Kong	141.379	2)	Hong Kong	2.389	2)	Hong Kong	26.747
3)	Shanghai	112.869	3)	Singapore	1.768	3)	Shanghai	22.040
4)	Wuhan	110.955	4)	Shanghai	1.146	4)	Singapore	20.823
5)	Singapore	108.419	5)	Wuhan	1.056	5)	Wuhan	20.652
6)	Nanjing	107.980	6)	Nanjing	1.045	6)	Cambridge	19.697
7)	Redmond	106.242	7)	Sydney	0.924	7)	London	19.672
8)	London	91.788	8)	Shenzhen	0.808	8)	Nanjing	19.465
9)	Xi’an	91.212	9)	Xi’an	0.732	9)	Sydney	17.162
10)	Mountain View	87.662	10)	Munich	0.662	10)	New York	15.131
11)	Munich	82.449	11)	Pittsburgh	0.601	11)	Xi’an	13.455
12)	Stanford	78.247	12)	Hangzhou	0.596	12)	Hangzhou	12.465
13)	Pittsburgh	77.227	13)	Harbin	0.561	13)	Pittsburgh	12.323
14)	Seoul	74.561	14)	Redmond	0.535	14)	Munich	11.854
15)	Berkeley	72.934	15)	Hefei	0.515	15)	Redmond	11.586
16)	Cambridge	72.545	16)	Taipei	0.505	16)	Boston	11.197
17)	Chengdu	60.808	17)	Cambridge	0.500	17)	Chengdu	11.091
18)	Hangzhou	60.071	18)	Boston	0.495	18)	Paris	10.475
19)	Sydney	59.025	19)	Chapel Hill	0.470	19)	Melbourne	10.268
20)	Paris	56.672	20)	Paris	0.465	20)	Los Angeles	10.268
21)	Oxford	56.323	21)	London	0.465	21)	Guangzhou	10.071
22)	New York	54.798	22)	Seoul	0.465	22)	Hefei	9.288
23)	Changsha	51.955	23)	Guangzhou	0.465	23)	Stanford	8.576
24)	Harbin	50.500	24)	Tokyo	0.455	24)	Shenzhen	8.535
25)	Los Angeles	48.561	25)	Zurich	0.434	25)	Seoul	8.333
26)	Toronto	48.177	26)	Chengdu	0.404	26)	Harbin	8.318
27)	Hefei	47.162	27)	Melbourne	0.379	27)	Philadelphia	7.722
28)	Boston	45.586	28)	New York	0.348	28)	Changsha	7.444
29)	Tokyo	43.773	29)	Seattle	0.348	29)	Mountain View	7.071
30)	Zurich	42.747	30)	Washington	0.343	30)	Tianjin	7.000
31)	Seattle	40.293	31)	Philadelphia	0.338	31)	Seattle	6.990
32)	Guangzhou	39.187	32)	Canberra	0.333	32)	Berkeley	6.596
33)	Tehran	38.828	33)	Toronto	0.318	33)	Toronto	6.566
34)	Montreal	38.505	34)	Tianjin	0.318	34)	Brisbane	6.460
35)	Tianjin	37.965	35)	Stanford	0.308	35)	Washington	6.111
36)	Dalian	35.677	36)	Dalian	0.303	36)	Canberra	5.970
37)	Chongqing	33.187	37)	Armonk	0.293	37)	Baltimore	5.687
38)	Menlo Park	31.242	38)	Los Angeles	0.293	38)	Montreal	5.672
39)	Taipei	30.278	39)	Ann Arbor	0.273	39)	Tokyo	5.520
40)	Forest Home	28.763	40)	Tehran	0.268	40)	Urbana	5.237

Supplementary Table 5: City ranking according to **closeness centrality** in the citation, migration, and collaboration networks. The color indicates whether the city is east (red) or west (blue).

Citation network			Migration network			Collaboration network		
1)	Beijing	297.676	1)	Beijing	4.573	1)	Beijing	82.779
2)	Redmond	274.693	2)	Hong Kong	4.272	2)	Hong Kong	63.264
3)	Hong Kong	267.166	3)	Singapore	3.676	3)	Shanghai	57.495
4)	Mountain View	250.455	4)	Shenzhen	3.399	4)	Wuhan	57.419
5)	Singapore	237.563	5)	Wuhan	3.332	5)	Nanjing	55.251
6)	Berkeley	225.990	6)	Shanghai	2.903	6)	Singapore	54.270
7)	Stanford	223.877	7)	Sydney	2.805	7)	Xi'an	48.936
8)	Wuhan	220.923	8)	Boston	2.663	8)	Sydney	47.068
9)	Nanjing	208.613	9)	Guangzhou	2.554	9)	Hefei	46.693
10)	Xi'an	207.847	10)	Xi'an	2.454	10)	Hangzhou	45.781
11)	Shanghai	205.709	11)	Harbin	2.388	11)	Chengdu	45.532
12)	Oxford	200.175	12)	Los Angeles	2.352	12)	Tianjin	45.324
13)	London	200.035	13)	Nanjing	2.302	13)	Redmond	45.030
14)	Pittsburgh	196.284	14)	Hangzhou	2.290	14)	Cambridge	44.497
15)	Munich	188.555	15)	Redmond	2.268	15)	Guangzhou	42.976
16)	Sydney	174.790	16)	Tokyo	2.244	16)	London	42.605
17)	Hangzhou	174.704	17)	Hefei	2.236	17)	Harbin	41.721
18)	Cambridge	173.723	18)	Tianjin	2.229	18)	Changsha	41.347
19)	Seoul	172.565	19)	Washington	2.143	19)	Shenzhen	41.020
20)	New York	170.066	20)	Dalian	2.081	20)	Boston	38.976
21)	Chengdu	168.153	21)	San Jose	2.022	21)	Los Angeles	36.499
22)	Harbin	164.829	22)	Chapel Hill	1.996	22)	Pittsburgh	36.234
23)	Toronto	161.705	23)	Urbana	1.967	23)	Dalian	35.270
24)	Menlo Park	159.661	24)	Chengdu	1.861	24)	Jinan	34.000
25)	Seattle	159.477	25)	Taipei	1.856	25)	Canberra	32.432
26)	Los Angeles	154.639	26)	Changsha	1.855	26)	Melbourne	32.380
27)	Changsha	153.129	27)	Armonk	1.774	27)	Jeddah	31.788
28)	Boston	152.833	28)	Waterloo	1.734	28)	Chongqing	31.441
29)	Montreal	152.532	29)	Chicago	1.733	29)	Tokyo	30.284
30)	Forest Home	151.739	30)	Pittsburgh	1.725	30)	Brisbane	30.167
31)	Paris	150.073	31)	Berkeley	1.712	31)	New York	30.019
32)	Hefei	147.038	32)	Ann Arbor	1.676	32)	Oxford	28.917
33)	Zurich	146.493	33)	Paris	1.673	33)	Munich	28.342
34)	Dalian	141.453	34)	Seattle	1.649	34)	Berkeley	27.740
35)	Guangzhou	140.423	35)	Munich	1.645	35)	Philadelphia	27.662
36)	Urbana	138.763	36)	Jinzhou	1.609	36)	Cambridge	26.223
37)	Tehran	133.576	37)	Cambridge	1.575	37)	Atlanta	25.791
38)	San Diego	129.121	38)	Bethesda	1.575	38)	Mountain View	25.647
39)	Chongqing	128.537	39)	Stanford	1.521	39)	Paris	25.310
40)	Rocquencourt	128.133	40)	Canberra	1.517	40)	Chicago	25.022

Supplementary Table 6: City ranking of top cities according to **PageRank centrality** in the citation, migration, and collaboration networks. The color indicates whether the city is east (red) or west (blue).

	Citation network			Migration network			Collaboration network	
1)	Beijing	0.0100118	1)	Beijing	0.0100062	1)	Beijing	0.0100084
2)	Shanghai	0.0100026	2)	Hong Kong	0.0100031	2)	London	0.0100029
3)	Wuhan	0.0100023	3)	Singapore	0.0100030	3)	Cambridge	0.0100022
4)	Nanjing	0.0100022	4)	Zurich	0.0100021	4)	Munich	0.0100021
5)	Hong Kong	0.0100017	5)	Sydney	0.0100017	5)	New York	0.0100017
6)	Xi'an	0.0100016	6)	Munich	0.0100017	6)	Shanghai	0.0100016
7)	Singapore	0.0100016	7)	Shanghai	0.0100016	7)	Paris	0.0100015
8)	Seoul	0.0100015	8)	Nanjing	0.0100013	8)	Nanjing	0.0100014
9)	London	0.0100014	9)	Seoul	0.0100013	9)	Hong Kong	0.0100014
10)	Munich	0.0100010	10)	Chengdu	0.0100012	10)	Singapore	0.0100013
11)	Chengdu	0.0100010	11)	Mountain View	0.0100011	11)	Pittsburgh	0.0100011
12)	Hangzhou	0.0100008	12)	Boston	0.0100011	12)	Wuhan	0.0100011
13)	Changsha	0.0100007	13)	Xi'an	0.0100010	13)	Sydney	0.0100010
14)	Paris	0.0100007	14)	Toronto	0.0100010	14)	Boston	0.0100008
15)	Cambridge	0.0100006	15)	Seattle	0.0100010	15)	Washington	0.0100008
16)	Sydney	0.0100006	16)	Hangzhou	0.0100010	16)	Seoul	0.0100007
17)	Pittsburgh	0.0100006	17)	Wuhan	0.0100008	17)	Redmond	0.0100005
18)	Hefei	0.0100005	18)	Rocquencourt	0.0100007	18)	Xi'an	0.0100005
19)	Harbin	0.0100005	19)	London	0.0100007	19)	Los Angeles	0.0100004
20)	Tehran	0.0100004	20)	Pittsburgh	0.0100006	20)	Stanford	0.0100004
21)	Tokyo	0.0100004	21)	Taipei	0.0100005	21)	Mountain View	0.0100004
22)	Boston	0.0100003	22)	Paris	0.0100004	22)	Chengdu	0.0100004
23)	Tianjin	0.0100003	23)	Redmond	0.0100004	23)	Montreal	0.0100004
24)	Guangzhou	0.0100003	24)	Madrid	0.0100004	24)	Melbourne	0.0100004
25)	Los Angeles	0.0100002	25)	Cambridge	0.0100003	25)	Toronto	0.0100003
26)	New York	0.0100002	26)	Washington	0.0100003	26)	Berkeley	0.0100003
27)	Redmond	0.0100001	27)	Stanford	0.0100003	27)	Seattle	0.0100003
28)	Chongqing	0.0100000	28)	New York	0.0100003	28)	Philadelphia	0.0100001
29)	Dalian	0.0100000	29)	Tokyo	0.0100003	29)	Armonk	0.0100001
30)	Taipei	0.0100000	30)	Shenzhen	0.0100003	30)	Hangzhou	0.0100000
31)	Zurich	0.0100000	31)	Cambridge	0.0100002	31)	Tehran	0.0100000
32)	Stanford	0.0099999	32)	Philadelphia	0.0100002	32)	Zurich	0.0100000
33)	Toronto	0.0099999	33)	Harbin	0.0100002	33)	Harbin	0.0100000
34)	Montreal	0.0099999	34)	Menlo Park	0.0100002	34)	Oxford	0.0100000
35)	Daejeon	0.0099998	35)	Melbourne	0.0100002	35)	Baltimore	0.0099999
36)	Shenzhen	0.0099998	36)	Jinan	0.0100000	36)	Urbana	0.0099999
37)	Melbourne	0.0099998	37)	Guangzhou	0.0100000	37)	Amsterdam	0.0099999
38)	Jinan	0.0099998	38)	Canberra	0.0099999	38)	Chicago	0.0099998
39)	Philadelphia	0.0099998	39)	Armonk	0.0099999	39)	Ann Arbor	0.0099998
40)	Mountain View	0.0099998	40)	Chapel Hill	0.0099998	40)	Tianjin	0.0099998

Supplementary Table 7: City ranking according to **independent cascade-based influence** in the citation, migration, and collaboration networks. The color indicates whether the city is east (red) or west (blue).

	Citation network			Migration network			Collaboration network	
1)	Beijing	3.092	1)	Beijing	2.742	1)	Beijing	5.993
2)	Hong Kong	2.616	2)	Hong Kong	2.023	2)	Hong Kong	3.909
3)	Redmond	2.565	3)	Singapore	1.667	3)	Shanghai	3.009
4)	Mountain View	2.175	4)	Wuhan	1.299	4)	Singapore	2.926
5)	Stanford	1.804	5)	Shanghai	1.126	5)	Wuhan	2.841
6)	Berkeley	1.793	6)	Sydney	0.843	6)	Nanjing	2.690
7)	Singapore	1.748	7)	Nanjing	0.794	7)	Sydney	2.291
8)	London	1.403	8)	Xi'an	0.726	8)	Cambridge	2.195
9)	Pittsburgh	1.381	9)	Shenzhen	0.705	9)	London	2.139
10)	Munich	1.357	10)	Harbin	0.628	10)	Hangzhou	1.908
11)	Oxford	1.355	11)	Hefei	0.491	11)	Xi'an	1.788
12)	Wuhan	1.293	12)	Guangzhou	0.476	12)	Redmond	1.786
13)	Nanjing	1.264	13)	Boston	0.476	13)	Guangzhou	1.623
14)	Cambridge	1.259	14)	Munich	0.468	14)	Chengdu	1.597
15)	Shanghai	1.224	15)	Pittsburgh	0.467	15)	Pittsburgh	1.555
16)	Xi'an	1.128	16)	Tianjin	0.427	16)	New York	1.535
17)	New York	1.033	17)	Paris	0.426	17)	Hefei	1.479
18)	Toronto	0.946	18)	Redmond	0.411	18)	Boston	1.472
19)	Seoul	0.915	19)	Chapel Hill	0.405	19)	Shenzhen	1.464
20)	Sydney	0.870	20)	Tokyo	0.404	20)	Los Angeles	1.409
21)	Seattle	0.841	21)	Taipei	0.398	21)	Harbin	1.280
22)	Los Angeles	0.802	22)	Cambridge	0.378	22)	Melbourne	1.260
23)	Paris	0.800	23)	Dalian	0.378	23)	Changsha	1.213
24)	Zurich	0.739	24)	Hangzhou	0.370	24)	Tianjin	1.165
25)	Montreal	0.736	25)	Washington	0.355	25)	Munich	1.151
26)	Menlo Park	0.728	26)	Chengdu	0.351	26)	Paris	1.035
27)	Hangzhou	0.714	27)	Los Angeles	0.344	27)	Seoul	1.012
28)	Forest Home	0.702	28)	Canberra	0.326	28)	Philadelphia	0.991
29)	Chengdu	0.697	29)	Seoul	0.308	29)	Stanford	0.964
30)	Harbin	0.656	30)	London	0.305	30)	Brisbane	0.915
31)	Boston	0.627	31)	Brisbane	0.286	31)	Dalian	0.893
32)	Changsha	0.569	32)	Changsha	0.274	32)	Seattle	0.799
33)	Rocquencourt	0.530	33)	Armonk	0.262	33)	Atlanta	0.792
34)	Cambridge	0.500	34)	Stanford	0.260	34)	Tokyo	0.786
35)	Urbana	0.498	35)	Zurich	0.256	35)	Berkeley	0.776
36)	Chapel Hill	0.495	36)	Philadelphia	0.240	36)	Toronto	0.741
37)	Tehran	0.478	37)	Tehran	0.234	37)	Canberra	0.710
38)	Hefei	0.475	38)	Ann Arbor	0.233	38)	Mountain View	0.707
39)	Dalian	0.470	39)	Waterloo	0.227	39)	Chongqing	0.699
40)	Tokyo	0.459	40)	Melbourne	0.227	40)	Urbana	0.679

Supplementary Table 8: City ranking according to **linear threshold-based influence** in the citation, migration, and collaboration networks. The color indicates whether the city is east (red) or west (blue).

	Citation network			Migration network			Collaboration network	
1)	Beijing	41.889	1)	Beijing	57.262	1)	Beijing	54.064
2)	Mountain View	36.319	2)	Hong Kong	38.751	2)	Cambridge	25.304
3)	Redmond	35.302	3)	Singapore	33.334	3)	London	24.841
4)	Stanford	32.073	4)	Shanghai	25.074	4)	Hong Kong	23.770
5)	Berkeley	30.279	5)	Wuhan	22.822	5)	Singapore	20.273
6)	Hong Kong	26.967	6)	Nanjing	19.313	6)	New York	19.665
7)	Cambridge	25.458	7)	Sydney	17.561	7)	Shanghai	19.652
8)	Munich	25.009	8)	Xi'an	15.725	8)	Sydney	17.431
9)	Pittsburgh	24.233	9)	Munich	14.071	9)	Munich	16.645
10)	London	23.663	10)	Paris	13.408	10)	Wuhan	16.234
11)	Oxford	21.586	11)	Shenzhen	12.470	11)	Nanjing	15.821
12)	Singapore	19.076	12)	Harbin	12.153	12)	Pittsburgh	15.675
13)	New York	18.659	13)	Pittsburgh	12.002	13)	Redmond	14.643
14)	Toronto	17.637	14)	Boston	10.768	14)	Los Angeles	14.533
15)	Seattle	14.771	15)	Guangzhou	10.167	15)	Paris	13.003
16)	Paris	14.485	16)	Taipei	10.130	16)	Boston	12.900
17)	Zurich	13.402	17)	Cambridge	10.000	17)	Stanford	11.929
18)	Shanghai	13.272	18)	Tokyo	9.844	18)	Seoul	11.928
19)	Los Angeles	13.201	19)	Chengdu	9.796	19)	Philadelphia	11.165
20)	Montreal	13.050	20)	Hefei	9.285	20)	Hangzhou	11.108
21)	Menlo Park	12.780	21)	Seoul	9.259	21)	Melbourne	10.829
22)	Seoul	12.580	22)	Redmond	9.184	22)	Xi'an	9.978
23)	Nanjing	11.861	23)	Hangzhou	9.132	23)	Mountain View	9.617
24)	Wuhan	11.328	24)	Chapel Hill	8.788	24)	Seattle	9.591
25)	Forest Home	11.117	25)	Tehran	8.614	25)	Berkeley	9.002
26)	Sydney	10.615	26)	Washington	8.366	26)	Chengdu	8.751
27)	Xi'an	10.428	27)	Zurich	8.182	27)	Washington	8.508
28)	Cambridge	9.742	28)	London	7.857	28)	Montreal	8.488
29)	Rocquencourt	9.386	29)	Philadelphia	7.631	29)	Baltimore	8.215
30)	Urbana	8.578	30)	Ecublens	7.490	30)	Guangzhou	8.213
31)	Boston	8.347	31)	Canberra	7.390	31)	Toronto	8.152
32)	Princeton	8.064	32)	Dalian	7.074	32)	Urbana	7.687
33)	Chapel Hill	7.884	33)	Tianjin	6.956	33)	Atlanta	7.427
34)	Philadelphia	7.731	34)	Brisbane	6.850	34)	Houston	7.291
35)	Baltimore	7.491	35)	Armonk	6.636	35)	Harbin	7.190
36)	Freiburg im Breisgau	7.468	36)	Los Angeles	6.492	36)	Tokyo	7.046
37)	Hangzhou	7.302	37)	Toronto	6.112	37)	Brisbane	6.977
38)	Santa Clara	7.194	38)	Ann Arbor	6.104	38)	Hefei	6.925
39)	Armonk	7.050	39)	Waterloo	5.995	39)	Zurich	6.827
40)	Tokyo	6.866	40)	Stanford	5.939	40)	Oxford	6.800

Supplementary Figures

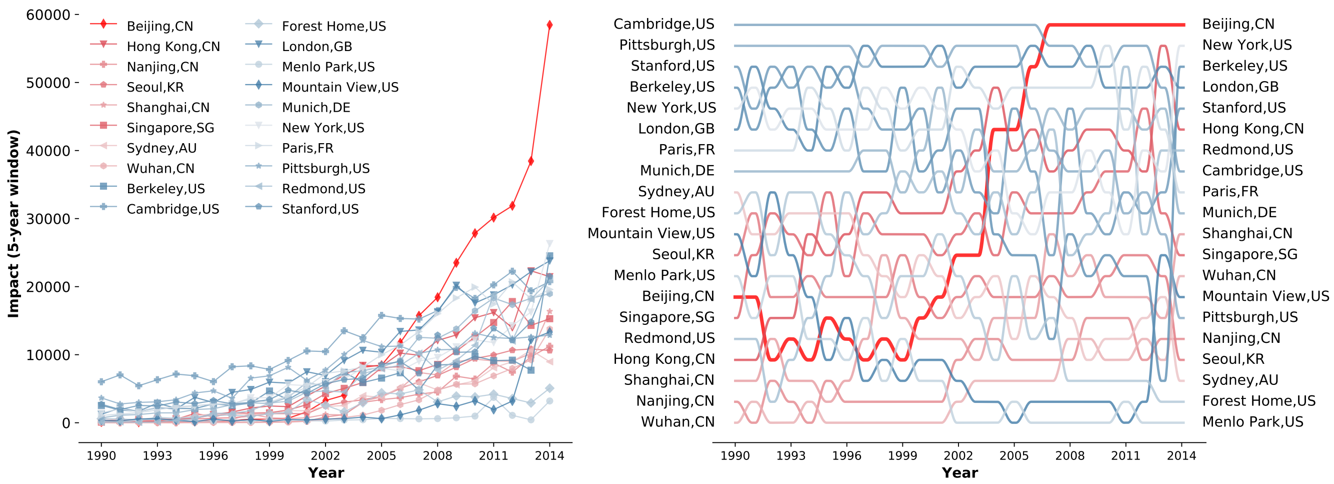

Supplementary Figure 1: **Comparing cities in terms of AI impact (similar to Figure 1a) but with a 5-year window instead of two**.

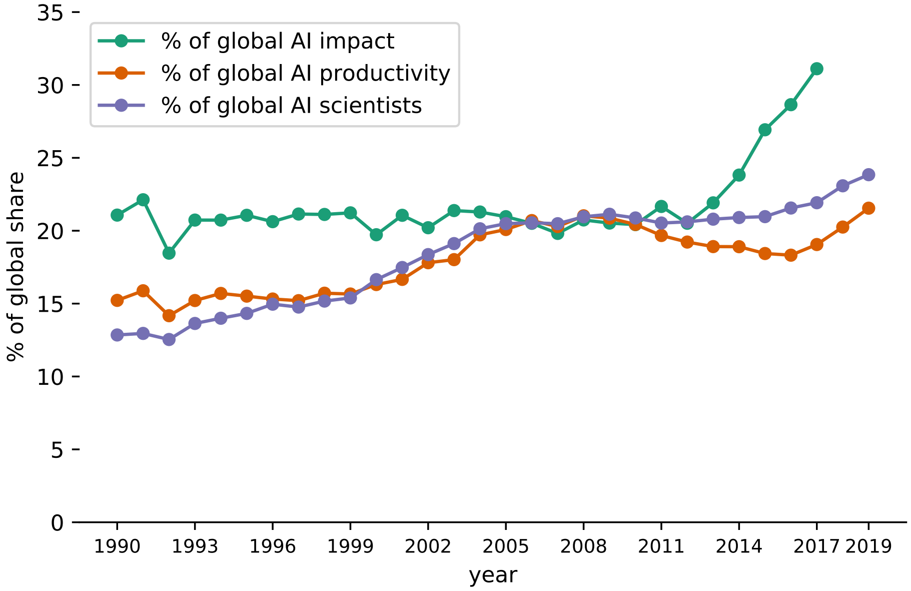

Supplementary Figure 2: **Top city's share of global AI research**. For each year, the figure depicts the top 20 cities' share of global AI impact, global AI productivity, and global AI scientists.

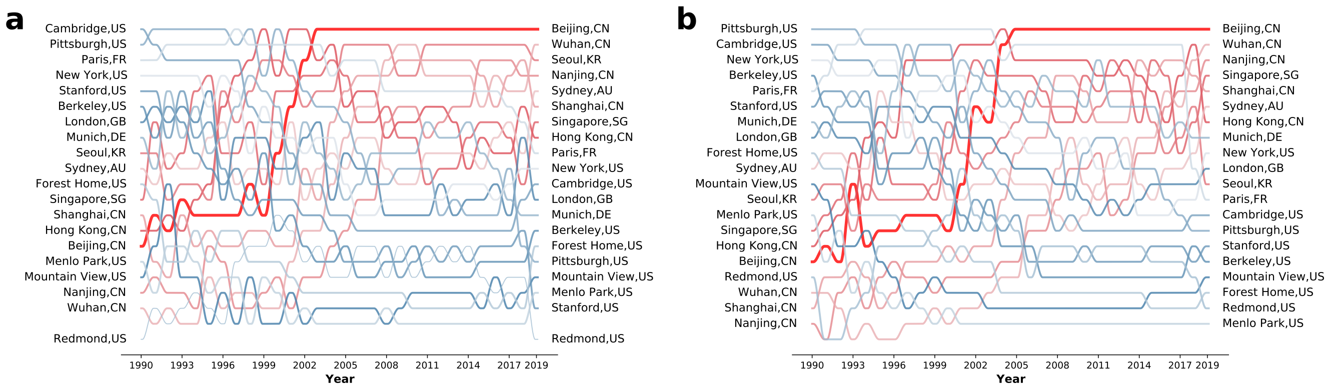

Supplementary Figure 3: (a) The most disruptive cities over time, (b) The most developmental cities over time.

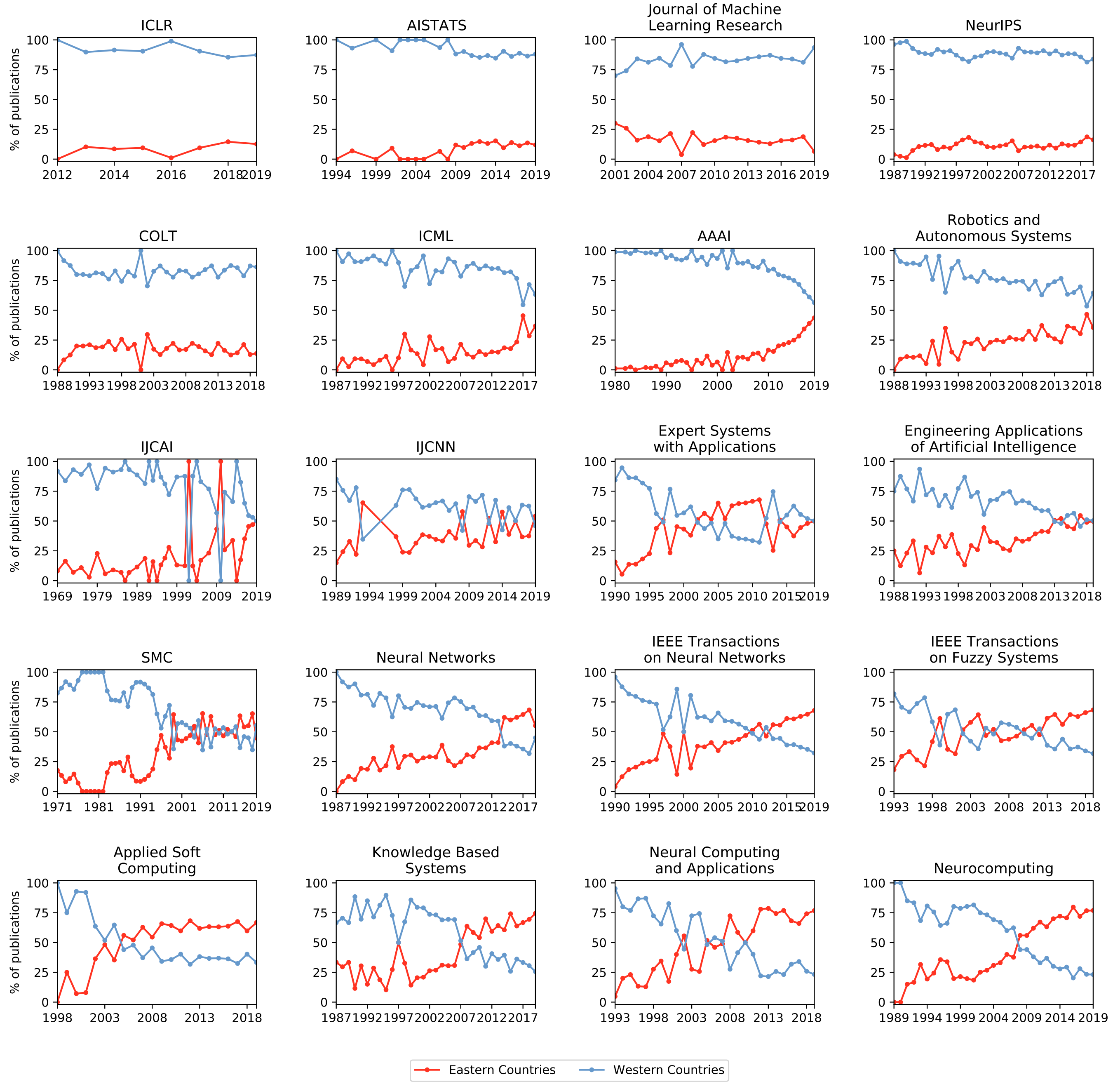

Supplementary Figure 4: The top 20 AI venues, ranked from left to right and top to bottom according to the share of papers produced by Western cities out of all papers published in the venue during the last five years. The first subfigure shows the venue where the share of Western cities is the largest, while the last subfigure shows the venue where the share of Eastern cities is the largest.

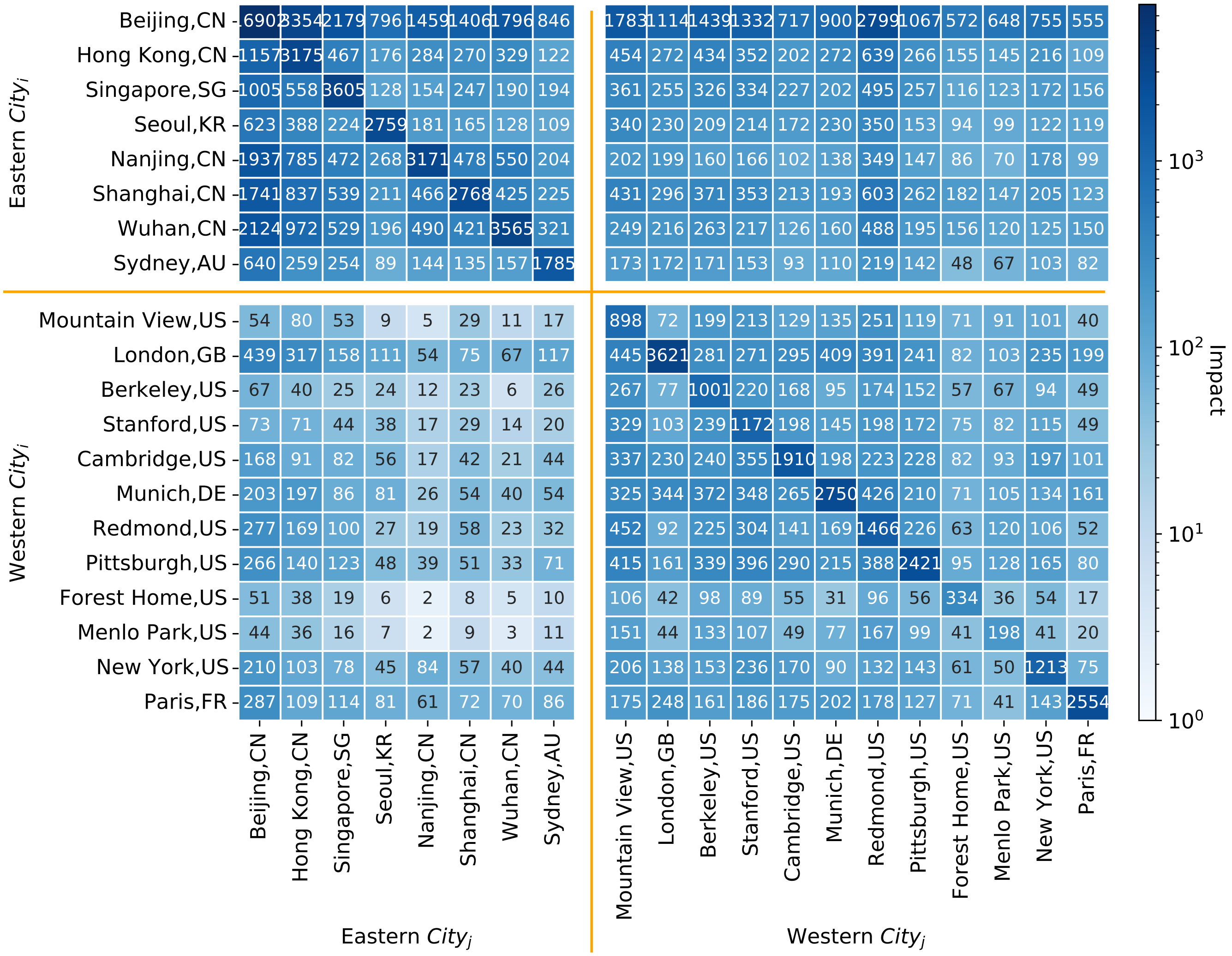

Supplementary Figure 5: **Figure 2a but with a logarithm scale**.

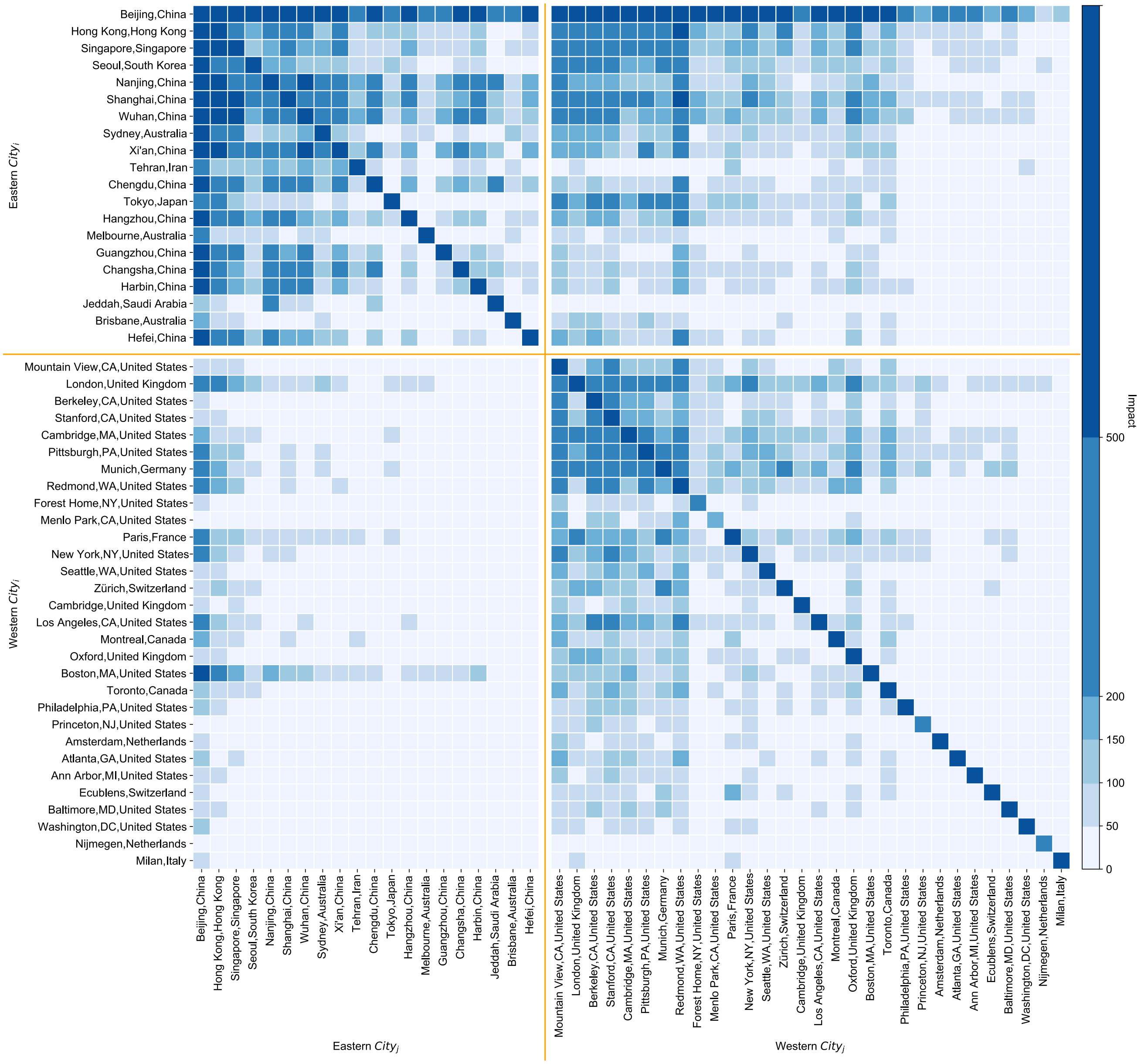

Supplementary Figure 6: **Pair-wise impact of 50 cities in terms of AI impact**. Similar to Figure 2a, except that we now consider the top 50 cities instead of the top 20.

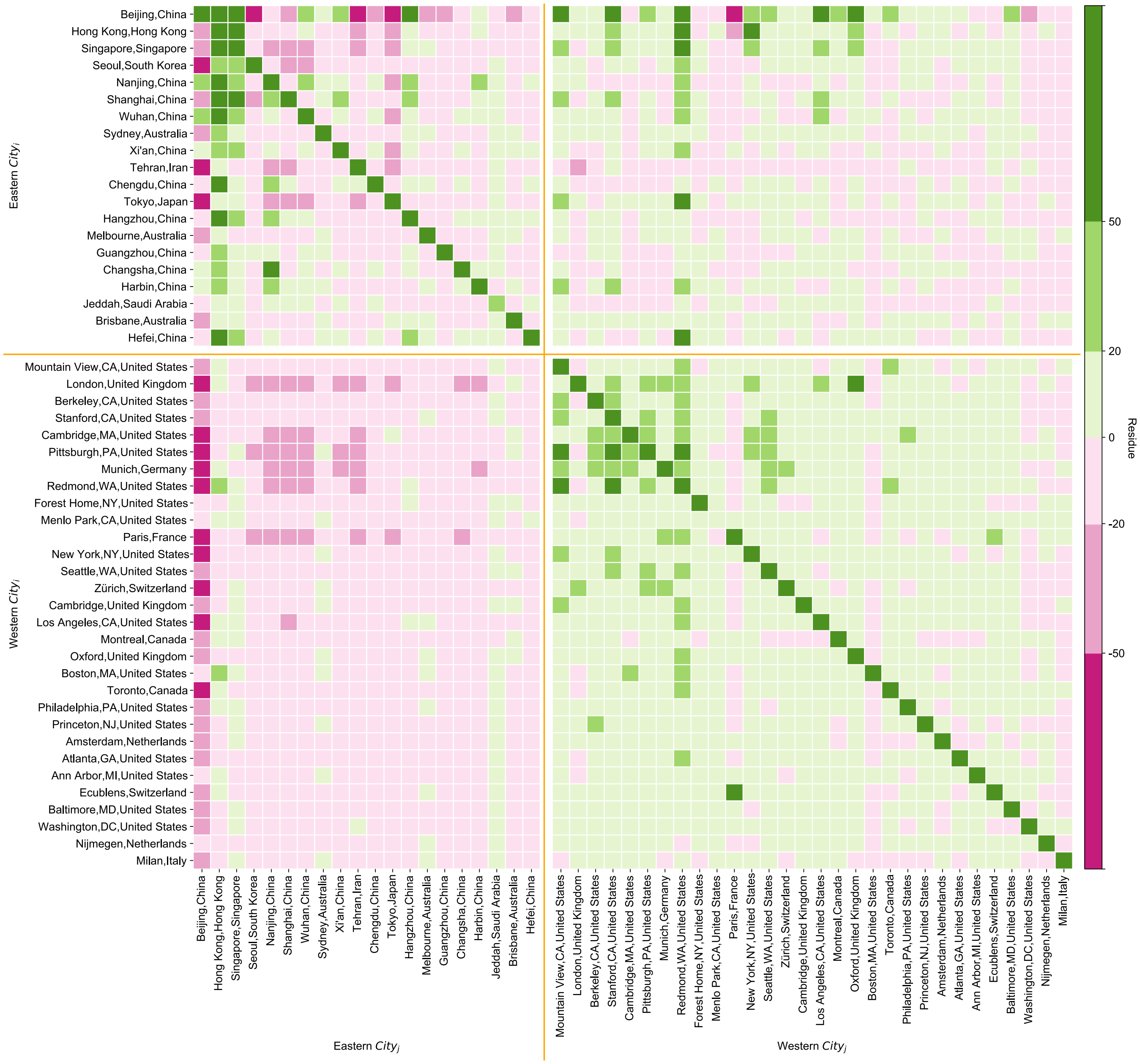

Supplementary Figure 7: **Pair-wise impact of 50 cities in terms of AI over-impact**. Similar to Figure 2b, except that we now consider the top 50 cities instead of the top 20.

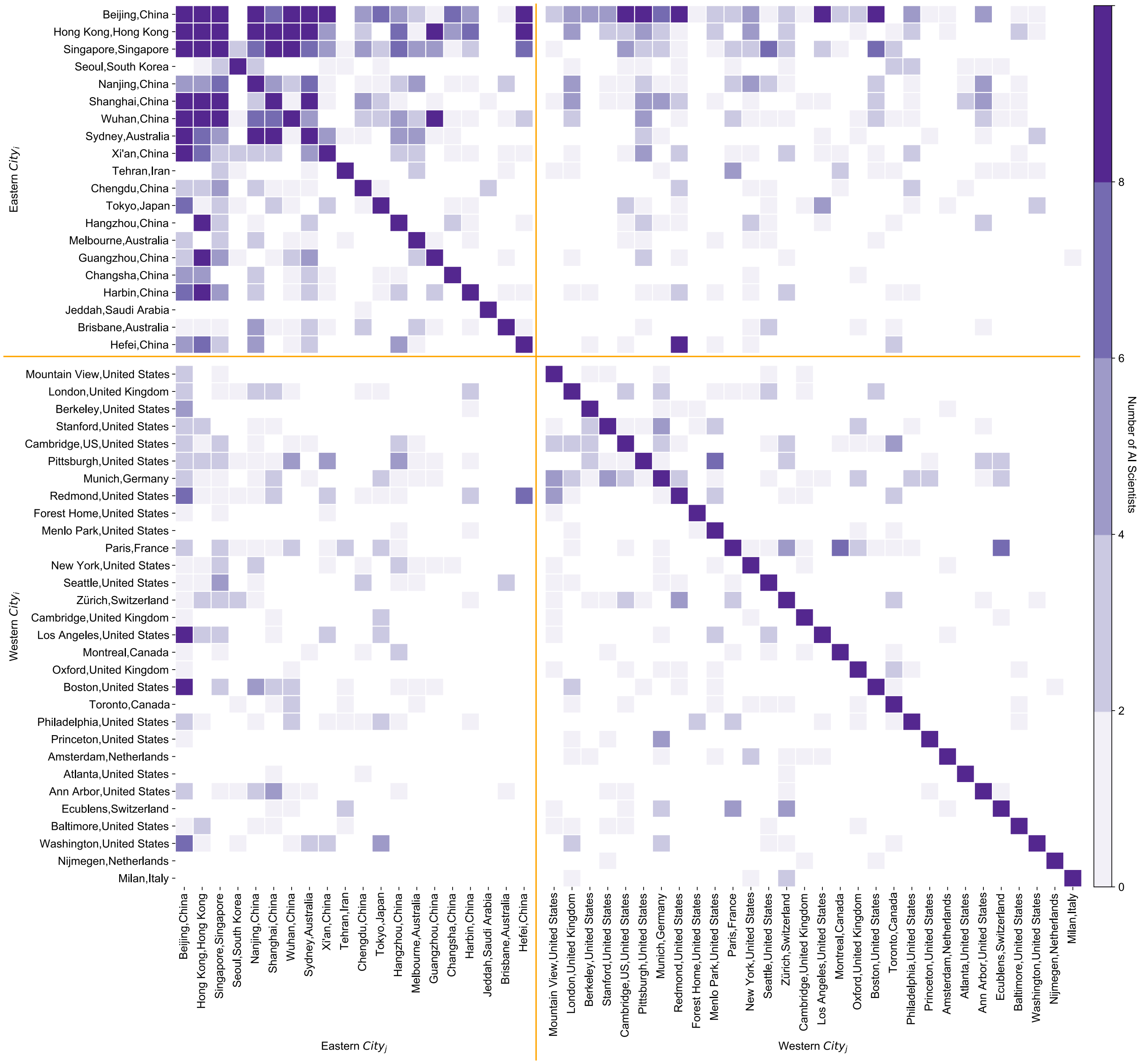

Supplementary Figure 8: **Migration between top 50 cities in terms of AI impact 2017**. Similar to Figure 2c, except that we now consider the top 20 cities instead of the top 20.

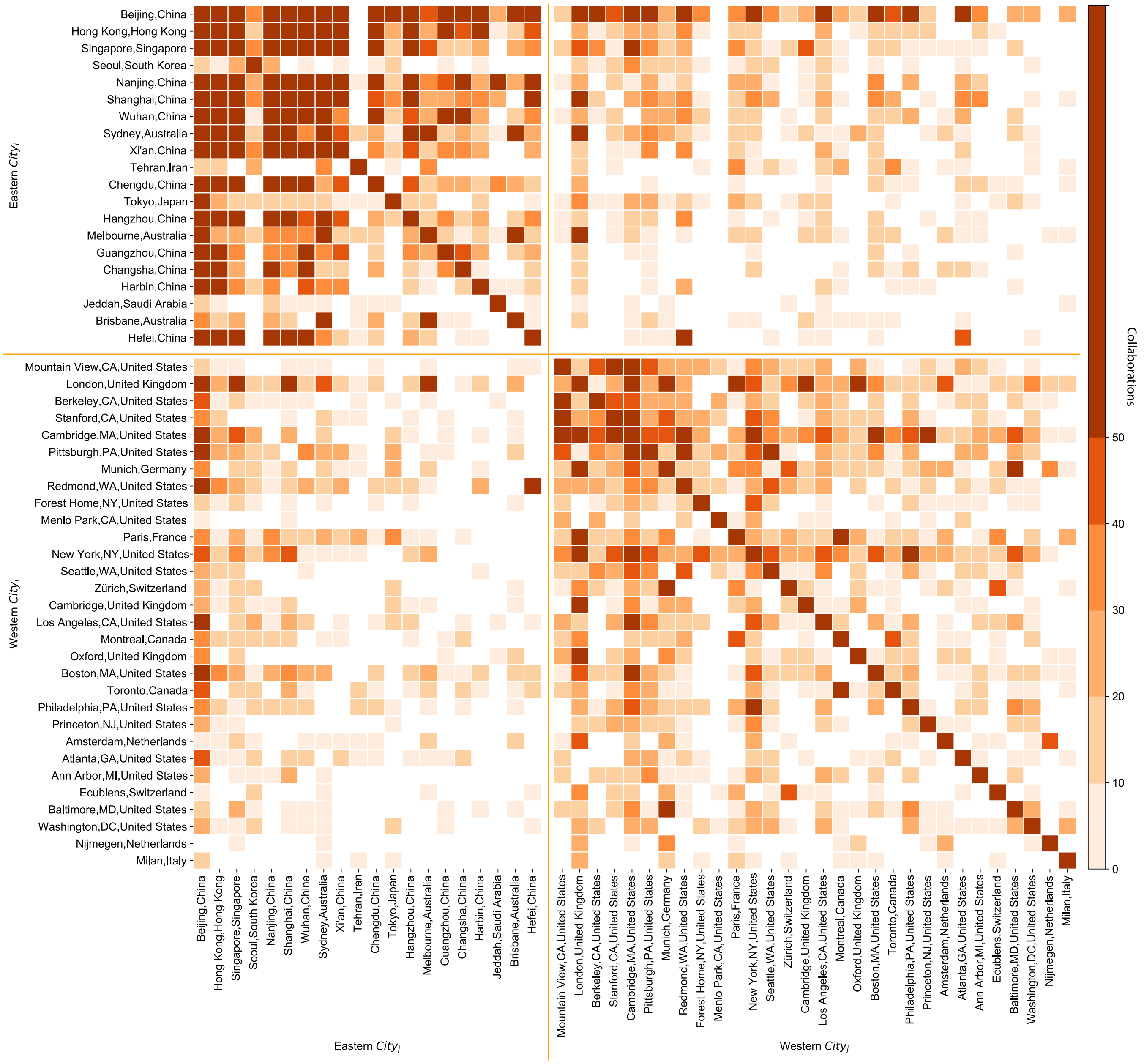

Supplementary Figure 9: **Collaboration between top 50 cities in terms of AI impact 2017**. Similar to Figure 2d, except that we now consider the top 50 cities instead of the top 20.

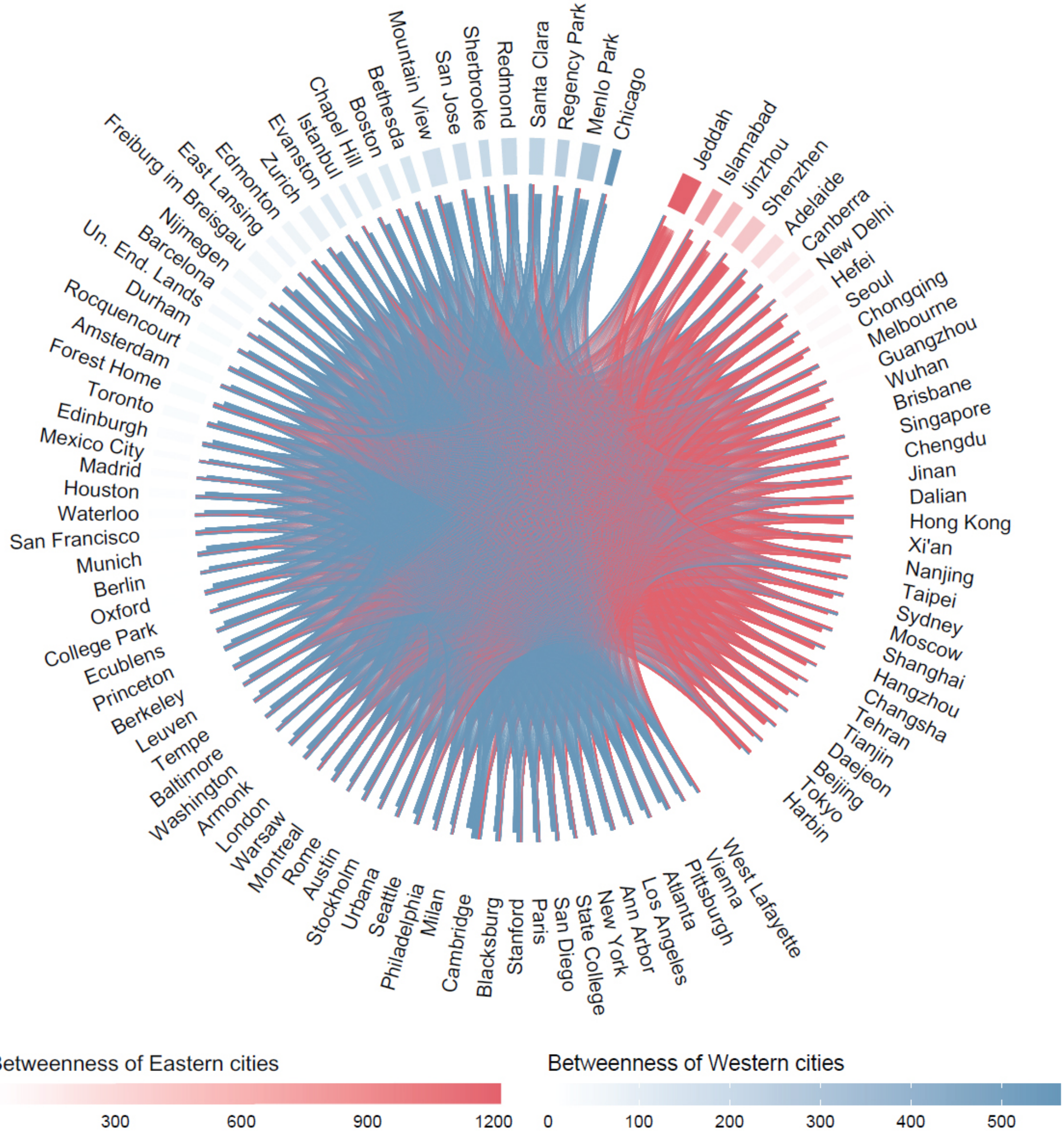

Supplementary Figure 10: **Betweenness centrality of different cities in the networks of normalized impact.** The chord diagram represents a normalized citation network, where the weight of the edge from city i to city j represents: (share of citations to j's AI papers that came from i's AI papers) divided by (j's share of global AI papers). Colors indicate whether the city is in the East (red) or the West (blue). For each city, the size of the corresponding arc (the portion of the outer ring) reflects the number of edges adjacent to the city, while the arc's color intensity reflects the city's betweenness. Cities are grouped into Eastern (right) and Western (left), and are sorted within each group according to betweenness. As for edges in the inner circle, the color indicates whether the edge points to a city in the East (red) or the West (blue), while the thickness indicates the edge's weight.

References

[1] Tinbergen, J. Shaping the world economy; suggestions for an international economic policy (1962).

[2] Anderson, J. E. The gravity model. *Annu. Rev. Econ.* **3**, 133–160 (2011).

[3] Chaney, T. The gravity equation in international trade: An explanation. *Journal of Political Economy* **126**, 150–177 (2018).

[4] Santos-Silva, J. & Tenreyro, S. The log of gravity. *The Review of Economics and statistics* **88**, 641–658 (2006).

[5] Anderson, J. E. & Van Wincoop, E. Gravity with gravitas: A solution to the border puzzle. *American economic review* **93**, 170–192 (2003).

[6] Mayer, T. & Zignago, S. Notes on cepii's distances measures: The geodist database (2011).

[7] Shaw, M. E. Group structure and the behavior of individuals in small groups. *The Journal of Psychology* **38**, 139–149 (1954).

[8] Beauchamp, M. A. An improved index of centrality. *Behavioral Science* **10**, 161–163 (1965).

[9] Page, L., Brin, S., Motwani, R. & Winograd, T. The pagerank citation ranking: Bringing order to the web. Tech. Rep., Stanford InfoLab (1999).

[10] Goldenberg, J., Libai, B. & Muller, E. Using complex systems analysis to advance marketing theory development: Modeling heterogeneity effects on new product growth through stochastic cellular automata. *Academy of Marketing Science Review* **9**, 1–18 (2001).

[11] Kempe, D., Kleinberg, J. & Tardos, É. Maximizing the spread of influence through a social network. In *Proceedings of the ninth ACM SIGKDD international conference on Knowledge discovery and data mining*, 137–146 (ACM, 2003).
